# Autophagy capacity and sub-mitochondrial heterogeneity shape Bnip3-induced mitophagy regulation of apoptosis

**DOI:** 10.1186/s12964-015-0115-9

**Published:** 2015-08-08

**Authors:** Sehyo Charley Choe, Anne Hamacher-Brady, Nathan Ryan Brady

**Affiliations:** Systems Biology of Cell Death Mechanisms, German Cancer Research Center (DKFZ), Heidelberg, Germany; Department of Surgery, Heidelberg University Hospital, Heidelberg, Germany; Bioquant, University of Heidelberg, Heidelberg, Germany; Lysosomal Systems Biology, German Cancer Research Center (DKFZ), Heidelberg, Germany

## Abstract

**Background:**

Mitochondria are key regulators of apoptosis. In response to stress, BH3-only proteins activate pro-apoptotic Bcl2 family proteins Bax and Bak, which induce mitochondrial outer membrane permeabilization (MOMP). While the large-scale mitochondrial release of pro-apoptotic proteins activates caspase-dependent cell death, a limited release results in sub-lethal caspase activation which promotes tumorigenesis. Mitochondrial autophagy (mitophagy) targets dysfunctional mitochondria for degradation by lysosomes, and undergoes extensive crosstalk with apoptosis signaling, but its influence on apoptosis remains undetermined. The BH3-only protein Bnip3 integrates apoptosis and mitophagy signaling at different signaling domains. Bnip3 inhibits pro-survival Bcl2 members via its BH3 domain and activates mitophagy through its LC3 Interacting Region (LIR), which is responsible for binding to autophagosomes. Previously, we have shown that Bnip3-activated mitophagy prior to apoptosis induction can reduce mitochondrial activation of caspases, suggesting that a reduction to mitochondrial levels may be pro-survival. An outstanding question is whether organelle dynamics and/or recently discovered subcellular variations of protein levels responsible for both MOMP sensitivity and crosstalk between apoptosis and mitophagy can influence the cellular apoptosis decision event. To that end, here we undertook a systems biology analysis of mitophagy-apoptosis crosstalk at the level of cellular mitochondrial populations.

**Results:**

Based on experimental findings, we developed a multi-scale, hybrid model with an individually adaptive mitochondrial population, whose actions are determined by protein levels, embedded in an agent-based model (ABM) for simulating subcellular dynamics and local feedback via reactive oxygen species signaling. Our model, supported by experimental evidence, identified an emergent regulatory structure within canonical apoptosis signaling. We show that the extent of mitophagy is determined by levels and spatial localization of autophagy capacity, and subcellular mitochondrial protein heterogeneities. Our model identifies mechanisms and conditions that alter the mitophagy decision within mitochondrial subpopulations to an extent sufficient to shape cellular outcome to apoptotic stimuli.

**Conclusion:**

Overall, our modeling approach provides means to suggest new experiments and implement findings at multiple scales in order to understand how network topologies and subcellular heterogeneities can influence signaling events at individual organelle level, and hence, determine the emergence of heterogeneity in cellular decisions due the actions of the collective intra-cellular population.

**Electronic supplementary material:**

The online version of this article (doi:10.1186/s12964-015-0115-9) contains supplementary material, which is available to authorized users.

## Background

Mitochondria are signaling hubs of cell survival and death signaling. Under normal conditions, mitochondria provide energy to the cell and regulate diverse processes [1]. Under apoptotic conditions, pro-death sensitizer BH3-only proteins bind and inhibit pro-survival Bcl2 members, while activator BH3-only proteins directly bind and activate Bax and Bak [2], triggering mitochondrial outer membrane permeabilization (MOMP). Consequently, MOMP releases pro-apoptotic Smac and cytochrome *c* into the cytosol, resulting in executioner caspase-mediated cell death within minutes [3]. Importantly, recent work challenges this paradigm, as cell lines and *in vivo* cells can recover following executioner caspase activation [4–6]. Furthermore, recent work has shown that sub-lethal executioner caspase activation is sufficient to stimulate DNA damage and resultant oncogenic transformation [4]. This process has now been shown to be triggered by activating MOMP in a subset of mitochondria following sub-lethal doses of apoptotic stimuli [7], suggesting that the MOMP capacity of a cell can determine an apoptotic cell death versus oncogenic transformation decision event.

Autophagy is a process requiring formation of autophagosomes, which are membrane-enclosed vesicles that capture cytosolic content, which fuse with, and then are degraded by lysosomes [8]. The specific mode of mitochondrial autophagy, mitophagy, is a quality control process for eliminating dysfunctional mitochondria via lysosomal degradation [9]. Bnip3 and Nix are BH3-only proteins which integrate apoptosis, autophagy, and mitophagy [10]. Bnip3 and Nix are autophagy receptors [11] containing nearly identical LC3-interacting regions (LIRs), which bind directly to LC3 proteins localized within autophagosomes, thereby engaging mitophagy [12–14]. *In vivo*, Bnip3-mediated mitophagy participates in mitochondrial homeostasis in liver of adult mice to avoid metabolic defects [15], its homologue Bnip3L/Nix regulates red blood cell maturation [16], and both regulate cardiac mitochondrial homeostasis [17].

Autophagy and mitophagy undergo complex regulatory crosstalk with apoptosis [18]. However, while mitophagy is well established to eliminate damaged mitochondria [19, 20], the function of mitophagy during apoptosis has not been resolved. First, MOMP execution occurs within minutes [21, 22], while mitophagy occurs progressively at a timescale of hours [13, 14, 23–25], even under constitutively-activated LIR conditions [14]. Second, following MOMP, apoptotic caspases inactivate the autophagy induction machinery [26–29], thus limiting autophagy induction capacity. Furthermore, co-perturbation of apoptosis and mitophagy, via expression of wild-type and mutant LIR-deficient autophagy receptors Bnip3 [12] and RNAi-mediated knockdown of FUNDC1 [23], did not differentially impact apoptosis induction. However, we recently reported that the Bnip3 LIR region is activated by serine phosphorylation, and by pre-activating mitophagy prior to Tumor Necrosis Factor (TNF) treatment, we observed a significant reduction to effector caspase activation [14]. We further reported that pro-survival Bcl-x_L_ positively regulates Bnip3 activation of mitochondrial sequestration.

The ensemble of the above findings suggest that the capacity of Bnip3 to reduce mitochondrial amplification of apoptosis is a function of the competition between Bnip3-mediated mitophagic and apoptotic activities, and moreover, a delay of MOMP activation is required for effective mitophagy. Therefore, in this study we sought to determine whether subcellular heterogeneities of protein or organelle content could significantly alter response sensitivities of individual mitochondria to Bnip3 signaling, and consequently, of mitochondrial populations as a whole to apoptosis and mitophagy pathway activations. To that end we focused on (1) intra-mitochondrial reactive oxidative species (ROS) signaling as a factor coordinating subcellular autophagy activity and mitochondrial apoptotic signaling, (2) Bcl2 member levels and distributions as factors underlying mitochondrial heterogeneity and (3) autophagy capacity in terms of levels and subcellular localization.

Mitochondrial ROS are generated during, and participate in, apoptosis signaling [30], autophagy [31] and *in vivo* mitophagy [32]. At the intra-cellular level, within minutes ROS-induced ROS release (RIRR) [33, 34] and ROS-dependent BH3-only protein-activated MOMP transmit as waves throughout mitochondrial populations [35]. Moreover, Bcl2 member levels and signaling heterogeneity can alter the mitochondrial apoptotic response. Following apoptotic stimuli the time to MOMP activation varies from cell-to-cell due to differences in Bcl2 member levels, resulting from variability in cellular rates of protein translation [36] and degradation [37]. Importantly, Bcl2 member heterogeneities present within mitochondrial populations can result in subcellular differences in MOMP sensitivities [38, 39].

To investigate the impact of local ROS signaling and Bcl2 member activities at the points of crosstalk on cell apoptotic decision, we used a hybrid, multi-scale model. An ordinary differential equation (ODE) model was used to simulate level-dependent dynamics in single mitochondria seeded with local information. The global, heterogeneous and adaptive behavior of mitochondrial populations was simulated within an agent-based environment.

Our findings illustrate the fundamental requirement for co-increased levels of LIR-active Bnip3 with pro-survival Bcl2 members to engage mitophagy to levels sufficient to alter apoptosis. Our model predicts a dependence of mitophagy activity on the level of autophagic vesicles (AV) within the cell, and more importantly, the necessity for AV proximity to mitochondria. Specifically, the model explores different AV spatial localizations, showing that a cell peripheral distribution is more efficient than peri-nuclear clustering, which we verify experimentally. Furthermore, simulations of delayed MOMP initiation by the activator BH3-only protein tBid suggest that the mitophagy pre-activation can de-sensitize the mitochondrial population to apoptosis signaling by reducing the level of cytochrome *c* release in a sub-population of mitochondria. Indeed, the key factor in this decrease is the variable response of the mitochondrial population. We show that increasing mitochondrial heterogeneity in Bax/Bcl2 levels gives rise to the emergence of sub-populations which can avoid MOMP activation and thus drive or amplify the capacity of mitophagy to negatively regulate apoptosis, and also, help to explain cell-to-cell variability. Further, we uncovered that heterogeneity of proteins acting at points of crosstalk at mitochondria differentially impact mitophagy potential, prior to, and following MOMP induction.

Overall, our findings offer comprehensive insights into the role and significance of heterogeneous subcellular behavior on the emergence of mitochondrial sub-populations, and their roles in shaping the cellular apoptotic response.

## Results

### Mitophagy and apoptosis pathway behavior for single mitochondrion

**Fig. 1:**
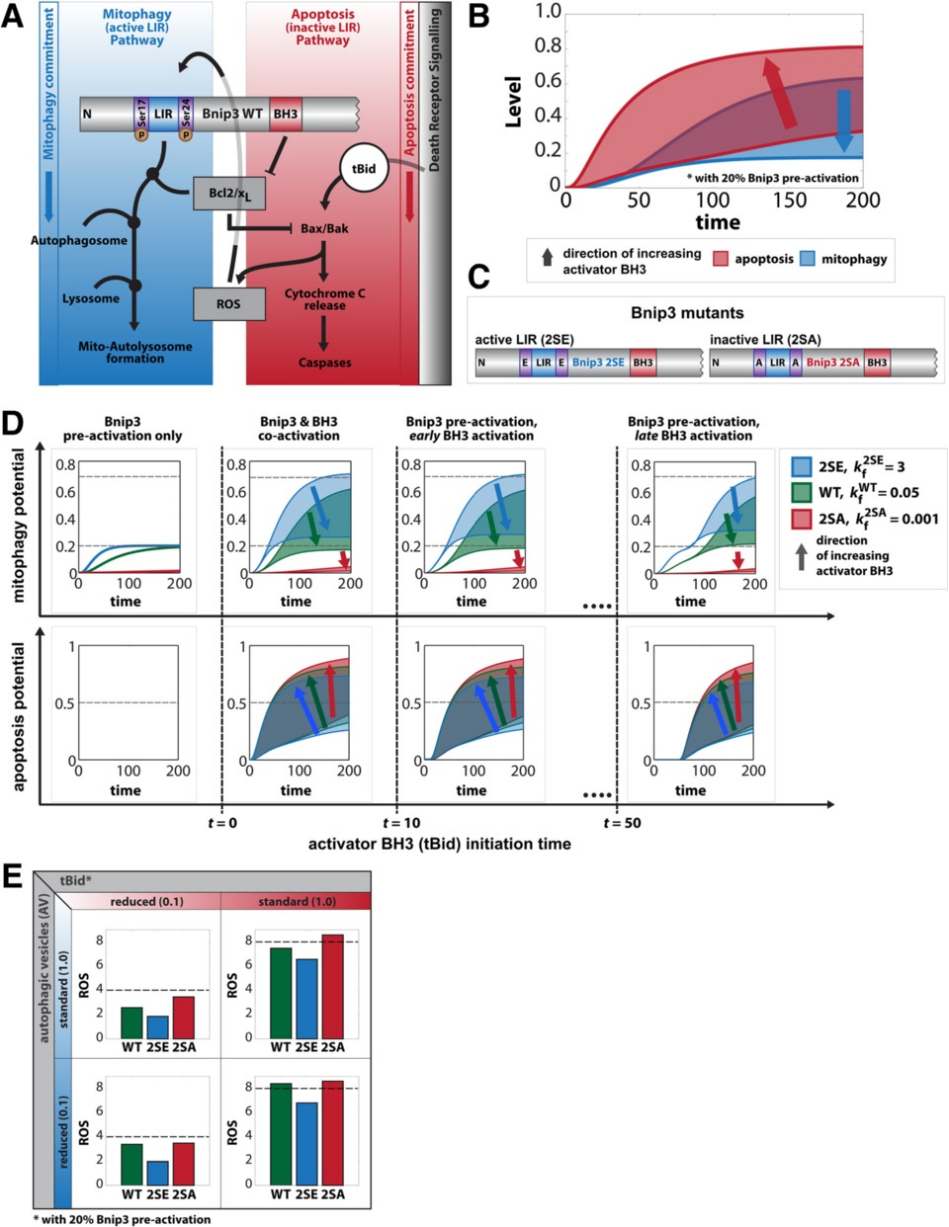
Single mitochondrion dynamics. **a** Illustration of Bnip3 dual-functionality due to LIR and BH3 domains. ROS and Bcl2/x_L_ (gray boxes) are points of crosstalk between two distinct branches: LIR-induced mitochondrial autophagy (mitophagy) pathway (blue) and apoptosis signaling by activator BH3 proteins (e.g. tBid), which induces Bax-mediated MOMP and cytochrome *c* release to induce caspase cascade (red). **b** Level values of the ODE species represent the mitophagy (blue) versus apoptosis (red) activity capacity for a mitochondrion. The shaded areas indicate range of activity as a function of increasing tBid activation (direction of arrows) and 20 % Bnip3 pre-activation. The overlap shows competition between both pathways via Bnip3. **c** Illustration of Bnip3 mutants with constitutively-active (2SE) and constitutively-inactive (2SA) LIR domain. **d** Scenarios of increasingly delayed timing of tBid activation (*t* = 0, 10, 50) for all mutants of Bnip3 with increasing tBid (direction of arrow) activation. **e** ROS production as a function of different tBid and autophagic vesicles (AV) level combinations for all three Bnip3 mutants

We first developed an ODE model for a single mitochondrion based on experimental findings in order to evaluate dynamic behavior stemming from Bnip3-mediated mitophagy and apoptosis signaling (Fig. 1a, Additional file 1: Figure S1). We concentrated on qualitative analysis by parameterizing our model with relative levels and constants (Additional file 2: Figure S2). In the apoptotic pathway (Fig. 1a, red), the BH3 domain of Bnip3 suppresses anti-apoptotic Bcl2 member function [40], acting as a sensitizer BH3 protein to tBid-mediated activation of Bax [41], and leads to ROS generation and caspase activation [21, 42]. In parallel, the mitophagy pathway (Fig. 1a, blue) is active when the phosphorylated LIR domain of Bnip3 binds autophagosomes, and Bcl-x_L_ positively regulates mitophagy [14]. Note, as ROS are central signaling messengers in apoptosis, autophagy and mitophagy [30–32], ROS was included as a prerequisite for activation of Bnip3 signaling [43].

A critical assumption of our ODE model is that LIR-mediated mitophagy and BH3-mediated Bax activation are branched pathways, with Bnip3 acting as an initiating signaling hub including multiple points of crosstalk. We tested this topology by expressing the constitutively-active LIR Bnip3-2SE mutant in Bax/Bak DKO cells [44]. Compared to control cells, we observed prominent sequestration of Bnip3-2SE-targeted mitochondria by autophagosomes (Additional file 3: Figure S3, yellow) confirming that the LIR-mediated mitophagy pathway is independent of MOMP activation.

Next, we established parameter sets, which qualitatively recapitulated our experimental findings by simulating combinations of low/high levels of Bcl2 and Bnip3, under conditions of increasing levels of tBid (Fig. 1b, Additional file 4: Figure S4). The mitophagy pathway was pre-initiated with a 20 % fraction of ROS-activated Bnip3, which we defined based on a relative increase in Bnip3 receptor binding to LC3B upon enforced LIR activation [14]. The simulations align with experimental observations of competition between mitophagic (blue shaded) and apoptotic (red shaded) pathways occurring only under conditions of high Bnip3 and high Bcl2 levels (Additional file 4: Figure S4, red box).

Given the fundamental importance of Bnip3 LIR activity on the extent of competition between both pathways, we simulated wild-type (WT) Bnip3 and constitutively-active LIR and inactivated-LIR mutants (hereafter 2SE, 2SA respectively; Fig. 1c). Specifically, under conditions of high Bcl2 and 20 % pre-activation of mitophagy, followed by subsequent activation of apoptosis by tBid, the model parameters were qualitatively calibrated to experimental observations. As such, in the absence of apoptosis signaling, the increased Bnip3 LIR activity of 2SE (Fig. 1d, first column, top, blue) showed a faster rate of mitophagy potential activation compared to WT (green), and while both reached maximal mitophagy potential, 2SE plateaued at a faster rate. On the other hand, the rate parameter for 2SA was chosen such that mitophagy potential remained almost negligible (red).

Subsequently, to simulate the experimental result that enhanced mitophagy activity, and/or delayed MOMP activation can reduce the capacity of mitochondria to amplify apoptosis [14], we tested co-activation of Bnip3 with increasing tBid levels (direction of arrows) and increasingly delayed the onset of tBid activation. The case of co-activation (Fig. 1d, second column) clearly showed an overlap of the mitophagy (top) and apoptosis (bottom) regions for WT and 2SE, indicative of competition between both pathways. Furthermore, as expected the 2SE mutant displayed mitophagy potential in excess of WT, while for the 2SA mutant there was no overlap of the mitophagy and apoptosis response regions, as expected due to its inactive LIR domain. For all three mutants, delaying the times of tBid activation (at *t* = 10 and 50) the same qualitative behavior in mitophagy and apoptosis response curves was observed as for tBid activation at *t* = 0, albeit with slightly reduced apoptosis response. Crucially, the impact of pre-activation of mitophagy on apoptosis potential was minimal, and not in agreement with experimental findings [14], suggesting the mitochondria do not function as a “well-mixed” system as assumed by ODEs but rather have inherent heterogeneity contributing towards interactions, and thus motivating our multi-agent approach presented below.

Notably, our model incorporates ROS production upon Bax activation, as it serves a dual function, and sits at a point of crosstalk (Fig. 1a). Under conditions of tBid expression, Bnip3 BH3 sensitizer activity induces ROS production, which positively feeds back to activate Bnip3. Furthermore, via co-activation of Bnip3 LIR activity, mitophagy can reduce apoptotic signaling. The ODE model simulations showed that under standard autophagy conditions and low tBid expression (Fig. 1e, upper left) the mitophagy-inactive 2SA mutant maximally increased ROS (red) compared to WT (green), while the LIR-active 2SE (blue) mutant maximally suppressed ROS production. Increasing tBid expression resulted in ROS amplification for all mutants, due to feedback from Bax activation (upper right). In contrast, under reduced autophagy conditions (lower panels) both low and high tBid conditions induced similar relative ROS production levels for WT and the 2SA mutant as before, albeit at higher levels, further indicating that autophagy capacity integrates with apoptosis signaling.

Overall, these results qualitatively reproduce our previous experimental findings [14] and the ODE model provides a basis for dynamic analysis of Bnip3 crosstalk between the apoptosis and mitophagy pathways. However, the model is insufficient to explain the observation of extensive variable response of a mitochondrial population in a cell, and hence, the impact of subcellular heterogeneity on cellular behavior.

### Multi-scale agent-based model for simulating mitochondrial population response

**Fig. 2:**
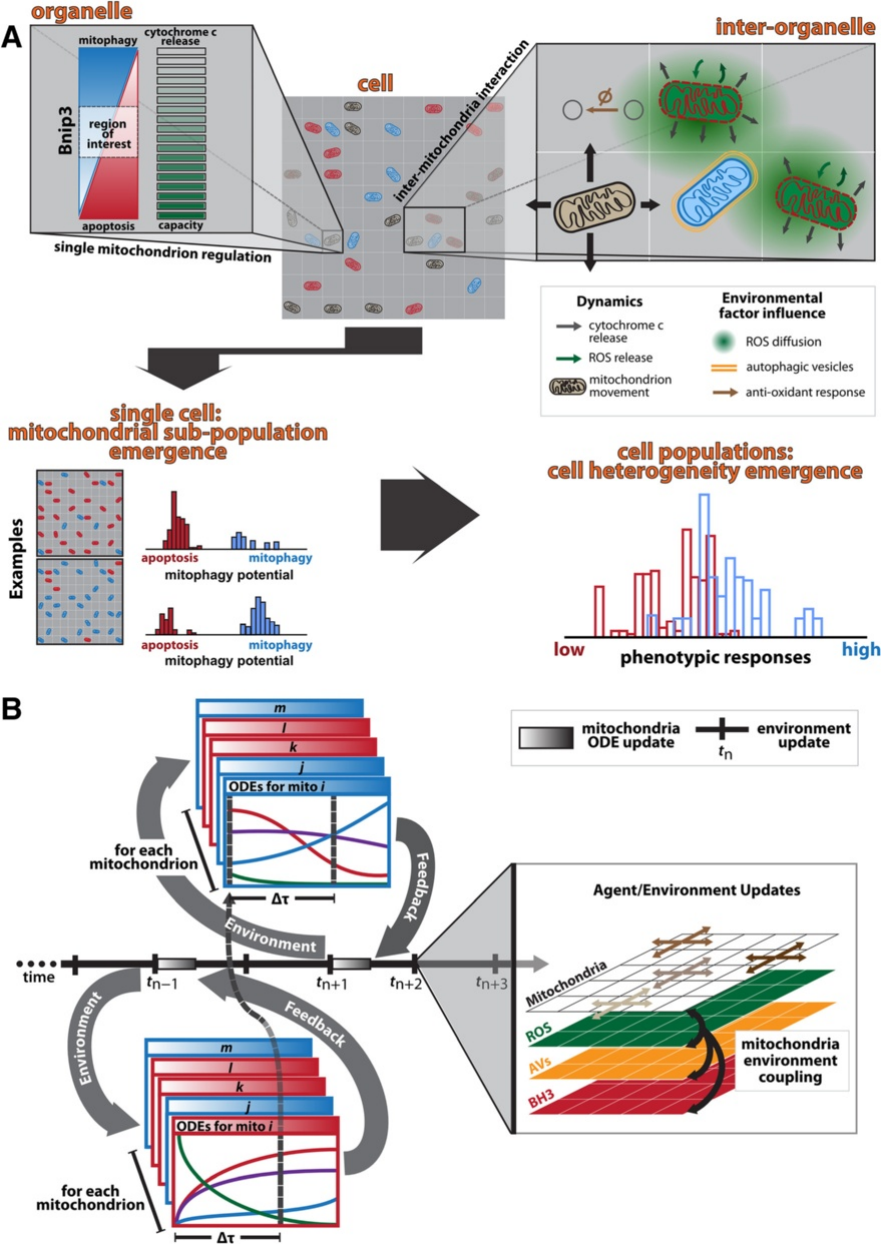
Embedding heterogeneous mitochondrial population in cell. **a** Schematic of multi-scale model embedding an ODE signaling network for each of the 100 mitochondria within an agent-based model (ABM). The full system size approximates a single cell (20 x 20 grid each assumed to be 1 μm x 1 μm to approximate a single mitochondrion), incorporating inter-organelle communication (ROS) by diffusion, as well as organelle dynamics by random motion, to explore impact of subcellular heterogeneities on cell-to-cell variability (see Additional file 2: Figure S2 for more details). **b** Schematic illustrating the timing rules for intra-, inter-mitochondrial and environmental updates. During mitochondrial ODE updates (every second agent-based time step) each mitochondrion’s ODEs are seeded with information from the local environment and previously stored content values, with any state changes evaluated at the end of the current time step. For agent-based time steps, environmental and mitochondrial movements are updated (see Additional file 2: Figure S2 for more details)

To that end, we developed a hybrid, multi-scale model in order to investigate how cellular behavior emerges from the collective action of a heterogeneous mitochondrial population, and characterize factors contributing to cell-to-cell apoptosis variability. We embedded one hundred, autonomous ODE models (one per mitochondrion) within an agent-based model (Fig. 2a) consisting of heterogeneous, adaptive layers of additional spatial biological information (see Materials and Methods; Additional file 2: Figure S2).

In contrast to the traditional fission/fusion model of mitochondrial networks and their impact on mitophagy events [45], in our model mitochondria were simulated as small units in order to approximate a fragmented state, according to experimental findings. Bnip3 induces mitochondrial fragmentation prior to mitophagy [46], and we determined that a co-increase of Bcl-x_L_ and Bnip3 promoted mitochondrial fragmentation compared to control (Additional file 5: Figure S5A), ruling out network interactions which could lead to mitophagy escape via mitochondrial fusion events [47]. Additionally, to characterize organelle mobility, we measured the dynamics of mitochondria and autophagosomes from time-lapse projections at one frame every 30 s for a total of 30 min (Additional file 5: Figure S5B). The widely-distributed colors in response to Bnip3 expression, indicates highly dynamic mitochondria, while the pronounced white regions for the autophagosome vesicles (AVs) suggests little mobility. Thus, we assigned random movement dynamics to mitochondria, and localized stationary AVs to an environmental layer.

At any given simulation time step, each mitochondrion’s behavior was a combination of its previous action history and the local information in its current position. Specifically, we implemented two time scales: mitochondrial ODE update and environmental update time steps (Fig. 2b). During a mitochondrial update step, each mitochondrion’s ODE model was seeded with the local environmental conditions (ROS, AV and tBid levels) and mitochondrial content stored from the previous time step. At the end of the ODE update time step, any output by the mitochondria (e.g. ROS, cytochrome *c*) was fed back onto the local environment, all intra-mitochondrial protein content was stored, and any state changes of the mitochondria determined. During environmental time steps, mitochondria randomly moved to nearest neighbors and all other environmental layers were updated assuming simple diffusion. These iterations continued until all mitochondria committed to a phenotype (Additional file 2: Figure S2).

This modeling approach enables the investigation of individual mitochondrion behavior and the collective actions of a population within a single cell on cell response, while many simulation runs uncover the extent of cell-to-cell variability.

### Mitochondrial population simulations reveal Bnip3 LIR impact to trigger cytochrome *c* release and AV location confers heterogeneity

First, to establish mitophagy induction behavior for Bnip3 WT and its mutants, we simulated Bnip3 pre-activation in a homogeneous mitochondrial population all with high Bnip3 and Bcl2 levels, randomly distributed in an environment of stationary autophagy vesicles (AVs), also randomly scattered throughout a cell. The pre-activation was due to a non-mitochondria-mediated, environmental ROS source, mimicking the conditions of a stressed cell [48], and were assumed to affect 20 % of Bnip3. At the single cell level, the potential of a mitochondrion to undergo mitophagy (‘mitophagy potential’), as well as its other factors such as cytochrome *c* release and ROS levels, were tracked over time. In addition, mitochondrion state phases of activation and competition between stimuli determined the final phenotype to which a mitochondrion committed. The collective final phenotypes of all mitochondria, as well as the total cytochrome *c* release, were recorded as an indication of final cell fate (see Additional file 2: Figure S2, light-green bounded box, for more details).

**Fig. 3:**
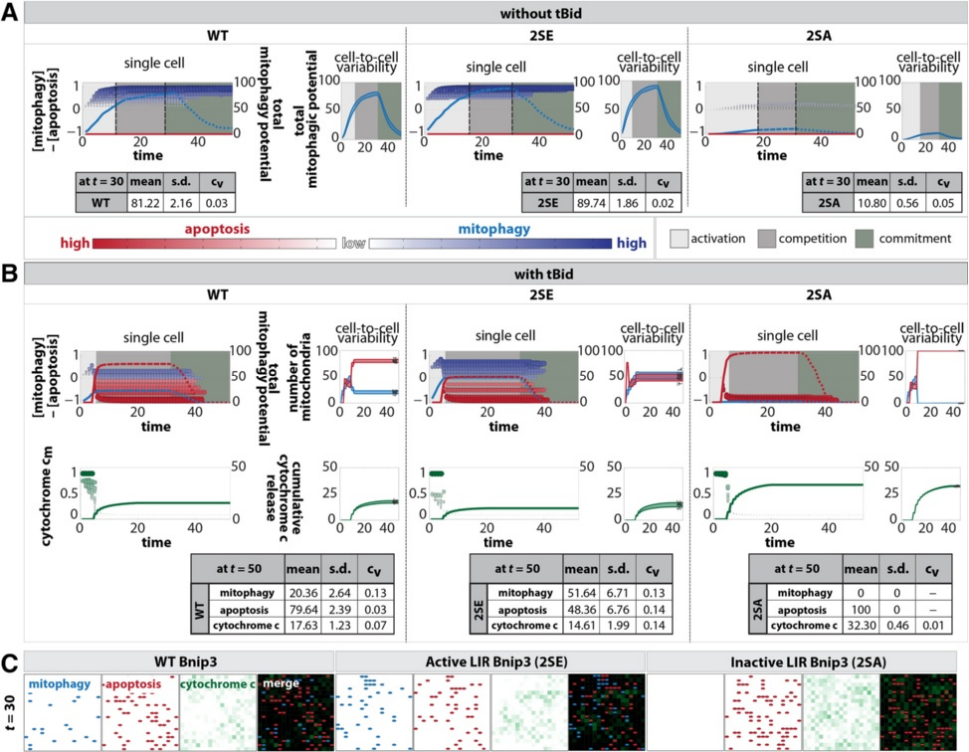
Impact of mitophagy/apoptosis signaling and ROS production on homogeneous mitochondrial population. Autophagic vesicles are randomly distributed to cover 20 % of cell surface (AV = 75) with a population of 100 mitochondria seeded with equal initial content (Bnip3 = 1, Bcl2 = 1, Bax = 1). All simulations are run for 100 time steps and sample size of 50 runs. **a** In absence of tBid activation and with 20 % pre-activation of Bnip3 LIR activity, scatter points (blue) track mitochondrial content individually, with size and color depth indicating level of mitophagy potential at every time step. Total mitophagic content in mitochondrial population (blue line) indicates the population response. The population exhibits three phases (insets): activation of signaling pathways (light gray), competition to commit to a phenotype (dark gray), and a committed phase during which phenotype is executed (olive). Runs show cell-to-cell variability (blue shaded area). Tables indicate statistics of total mitophagy potential after 30 time steps around the time of phenotype commitment, with sample size of 50 runs. **b** With tBid activation at *t* = 5 following 20 % pre-activation of Bnip3 LIR activity shows total apoptotic potential (red line) and total mitophagy potential (blue line). Scatter points track mitochondrial content individually showing mitophagy (blue) or apoptosis (red) potential at every time step. (Inset top) Final phenotype of all mitochondria shows size of mitophagy (blue) and apoptosis (red) sub-populations, and consequent cytochrome *c* release (green). Tables show statistics after 50 time steps, sampled over 50 runs. **c** Example simulation images (at *t* = 30) of 20 % pre-activation of Bnip3 followed by tBid activation (at *t* = 5), showing mitochondrial sub-populations with mitophagy (blue) or apoptosis (red) phenotypes and cytochrome *c* release environment layer (green); merged (black background)

To simulate mitophagy in a single cell, an initial level of AV = 75 was distributed to approximate a 20 % coverage of the total cell surface area. For a single cell, the scatter points (Figure 3a) represent the time-dependent evolution of each mitochondrion’s phenotype, with the size and color indicating the dominant phenotypes, either mitophagy (blue) or apoptosis (red). Notably, the agent-based simulation resulted in emergence of mitochondrial heterogeneity. The spread of the scatter points along the y-axis indicates the emergent variable response of the mitochondrial population to mitophagy activation. This variability arises from the heterogeneity in co-localization of mitochondria to AVs, as mitochondria in the vicinity of AVs are affected more potently, resulting in the variable degradation rate of the population (along x-axis). In comparison, an homogeneous distribution of AVs, where total AV content was spatially distributed equally, shows almost no variability in mitochondrial response (Additional file 6: Figure S6A), implicating the importance of heterogeneous AV localization.

As an indication of a cell’s mitochondrial population activity, the total mitophagy and apoptosis potential was overlaid (as blue and red lines respectively). Typical simulations showed three distinct phases of mitochondrial action and resulting cellular evolution (see ‘State definitions’ in Additional file 2: Figure S2). Firstly, the Bnip3 LIR pre-activation resulted in an ‘activation’ phase (Fig. 3a, solid blue line & light gray region in inset), where the phenotype of a mitochondrion developed. During the ‘competition’ phase (dashed blue line & dark gray region) mitochondria went through multiple cycles, continually adapting their individual states through feedback with the environment to determine their final phenotype. Finally, once a mitochondrion remained in a specific phenotype for 15 consecutive time steps (see *Methods*) it entered the permanent ‘committed’ phase (dotted blue line & olive region). Note, the downward slope of the total mitophagy potential and the disappearance of the scatter points during the committed phase are due to the degradation of the mitochondrial population.

In the absence of tBid activation (Fig. 3a), 2SE resulted in a higher and faster cellular commitment to mitophagy (blue) relative to WT, while the 2SA mutant only induced a very weak response. At the cell population level, the spread between the individual runs (inset, blue shaded area) indicates cell-to-cell variability in all phases. On average, at the maximal point prior to final mitochondrial commitment to mitophagy, 2SE increased mitophagy potential approximately 10 %, while 2SA reduced mitophagy potential by 90 % compared to WT (Fig. 3a, statistics table).

Subsequently, in order to investigate the impact of mitophagy pre-activation on altering the mitochondrial population response to apoptosis signaling, we repeated the simulations, followed by induction of tBid at relative time point *t* = 5. The combination of opposing stimuli resulted in a shortened activation phase (Fig. 3b) and prolonged competition phase (dashed lines). For WT, the increase in total apoptotic potential in mitochondria (red line) resulted in 4-fold more mitochondria with apoptosis phenotype than mitophagy. The increased LIR activity of the 2SE mutant reduced the susceptibility of the mitochondrial population to tBid activation, resulting in a mitochondrial population approximately equally split in both phenotypes, and a mitophagy phenotypic population 2.5-fold higher than WT. Conversely, the inactive LIR of the 2SA mutant is evident in the total mitochondrial population commitment to apoptosis. The role of LIR activity is further evident in the cytochrome *c* release levels. While the 2SE mutant reduced cytochrome *c* slightly compared to WT, the 2SA mutant doubled release, which was indicative of the sharp commitment of all mitochondria towards MOMP. Snapshots at time *t* = 30 illustrate how the phenotype of the mitochondrial population varied between the mutants, highlighting the consequence on cellular environment (Fig. 3c).

Together, these results demonstrate that a homogeneous mitochondrial population in a dynamic signaling environment evolves towards subpopulation heterogeneity, which underlies cell-to-cell variability. Our model suggests that AV spatial localization contributes to the emergence of mitochondrial heterogeneity. Further, our model intimates that Bnip3 LIR activity impact on subpopulations of mitochondria can alter cell-level canonical apoptosis signaling pathway.

### Environmental variable impact on mitophagy and subpopulation emergence

Next, we sought to understand additional factors contributing to emergence of mitochondrial subpopulations, and first focused on the role of ROS on regulating mitochondrial behavior. To perturb ROS production (Additional file 7: Figures S7A-S7C), we altered its secondary, non-mitochondrial source (green dashed lines). Relative to standard conditions (Additional file 7: Figure S7B) a 5-fold decrease in environmental ROS production reduced the number of mitophagic mitochondria 10-fold, due to the lack of Bnip3 activation (Additional file 7: Figure S7A). In contrast, a 4-fold increase in activation of ROS doubled the number of mitophagic mitochondria (Additional file 7: Figure S7C). Furthermore, a decreasing coefficient of variation for increasing ROS production rates suggests that ROS signaling dominates mitochondrial decisions. Correspondingly, total cytochrome *c* release was increased by reduced ROS production, and suppressed by increased ROS production, reflecting the role of ROS-promoted mitophagy on apoptosis capacity. Interestingly, while ROS production plays a crucial role on the level of mitophagy and cytochrome *c* release, the level of ROS degradation had a lesser impact (Additional file 7: Figures S7D and S7E respectively). Furthermore, this supports the function of ROS as a messenger of apoptotic signal between mitochondria, able to influence local ROS levels, creating a heterogeneous ROS environment, where the action of the dynamically moving mitochondria may be impacted locally. This is suggested in the mitochondrial population response upon tBid induction. Here, the initial homogenous mitochondrial population, even under homogeneous AV content, showed heterogeneity due to MOMP-mediated ROS release, which created local environmental heterogeneity to impact mitochondria in the vicinity (Additional file 6: Figure S6B, spread in red scatter). However, heterogeneity is only partially driven by ROS. Therefore, we examined the other main environmental variable, autophagy capacity, i.e. AV levels and localization.

### Access to AVs underlie mitophagy regulation and variable response

**Fig. 4:**
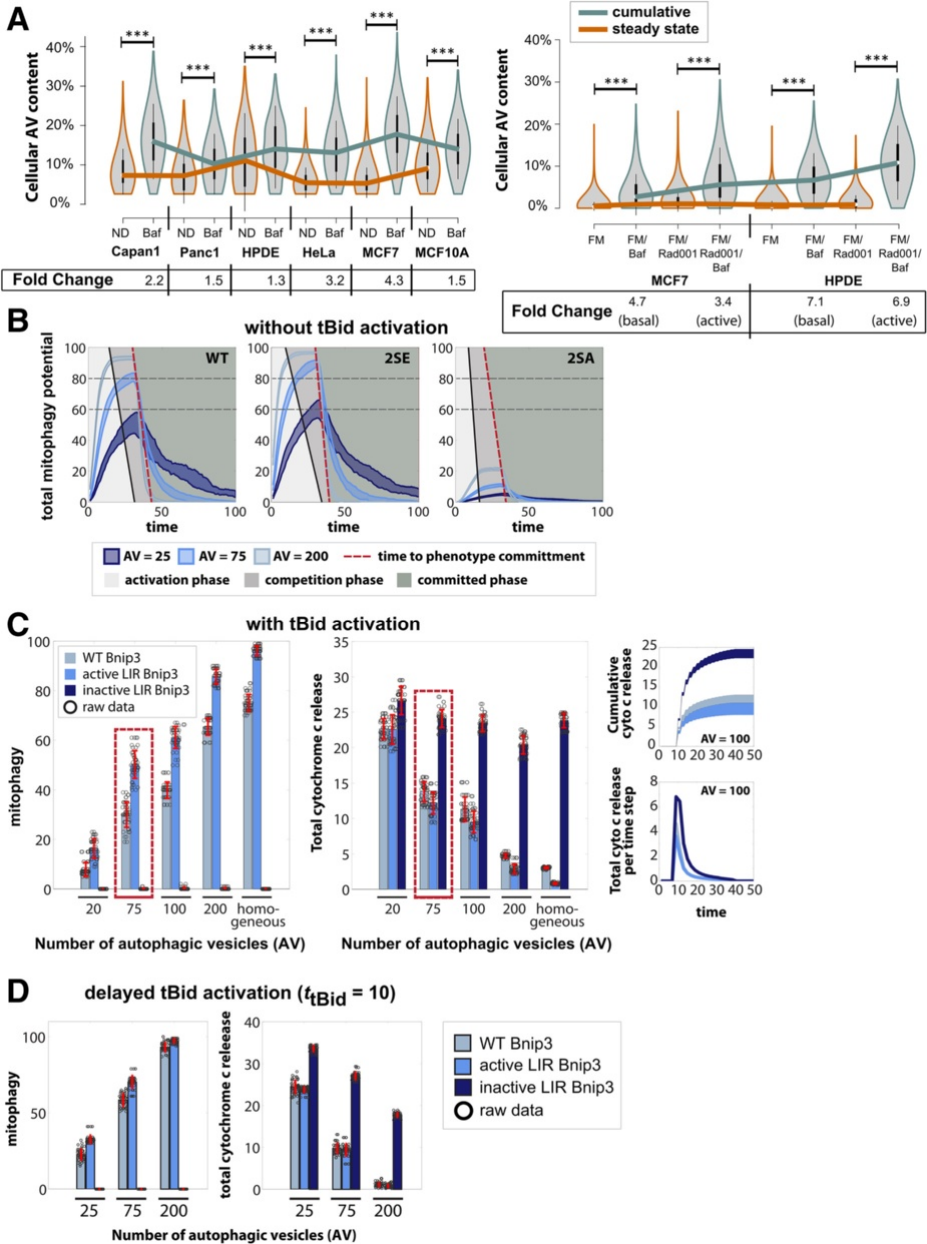
Impact of AV level on homogeneous mitochondrial population. **a** Imagestream X analysis of normalized GFP-LC3 AV intensities in cell populations under basal, activated and Bafilomycin A1 (Baf)-inhibited autophagy conditions for 3 h. Autophagic flux is reported as fold change, calculated from mean normalized steady state and cumulative (Baf-treated) GFP-LC3 AV intensities. (left) Experiments under conditions of autophagy activation by nutrient deprivation (ND), and ND with lysosomal inhibitor Baf (100 nM) treatment in cell lines: Capan1 and Panc1 (pancreatic cancer), HPDE (non-tumorgenic pancreatic epithelial), HeLa (ovarian cancer), MCF7 (breast cancer), MCF10A (non-tumorgenic breast epithelial). (right) Autophagy activation via treatment with mTOR inhibitor RAD001 (100 nM), and in RAD001 combined with Baf under conditions of full medium (FM). **b** Model simulations using similar fold changes in AV content as in (**a**), in absence of tBid activation for homogeneous mitochondrial population. Results indicate mitophagy activation rate during an “activation” phase (light gray region), beginning (solid black line) of a “competition” phase (dark gray region), and point of mitophagy phenotype commitment (dashed red line) for all Bnip3 mutants. Spread in curves for each condition indicates cell-to-cell variability. **c** (left) Total number of mitochondria in a cell committed to mitophagy as final phenotype after tBid activation (at *t* = 5) and 20 % Bnip3 pre-activation with increasing AV levels for all three Bnip3 mutants and (middle) corresponding total cytochrome *c* release. (inset, bottom) Total cytochrome *c* release per time step by all mitochondria and (inset, top) total cumulative cytochrome *c* release for Bnip3 WT (**d**) Impact of delayed tBid activation (at *t* = 10) on cellular mitophagic response and total cytochrome *c* release for increasing AV levels. All simulated conditions had sample size of 50 runs with scatter points indicating a single run

Different cell types likely have varying autophagy capacities, which may determine how AVs influence mitophagy induction. Hence, in order to explore the contribution of cellular autophagic capacity on the emergence of subcellular and cellular heterogeneity, we sought to systematically simulate different AV levels. To qualitatively model a physiological range of cellular autophagy capacity, we first experimentally measured autophagy flux in populations of cancer and non-cancer cell lines. Cells were submitted to 3 h of nutrient deprivation (ND), and ND in the presence of the lysosomal inhibitor Bafilomycin A1 (Baf) to measure flux [49]. Single cell analysis of autophagy in cell populations was performed with image-based flow cytometry analysis using Imagestream [14, 50]. As a measure of AV content, steady-state GFP-LC3 vesicles intensity was normalized to cumulative cell GFP-LC3 intensity, which allowed for direct comparison of population responses between cell types and conditions (Fig. 4a, Additional file 8: Figures S8A-B). The mean steady-state (orange traces) and cumulative (Baf-inhibited) AV content (olive traces) showed ranges of 1.3–4.3 fold differences between cell types. Similarly, we compared basal autophagy response by inducing autophagy with the mTOR inhibitor RAD001 under full medium (FM) conditions in breast cancer MCF7 cells and human pancreatic duct epithelial (HPDE) cells. Both cell types showed high autophagy flux after treatment with Baf and RAD001 showing approximately 3–7 fold increases.

Based on these fold changes, we next analyzed, at the cell population level, the impact of altering autophagy capacity. It should be noted that autophagy flux is rapid, with autophagosome turnover within minutes [51]. In contrast, sequestered mitochondria are degraded at a timescale of hours to days [13, 14, 23–25]. Therefore, to simplify our model, we did not allow for turnover of autophagosomes, but assumed that levels of autophagosomes represented different autophagy capacity states of the cell.

We simulated an approximate 3-fold decrease and increase in AV levels relative to standard AV content, with a range from low levels at 25 to maximal at 200, and a standard value of 75 (Fig. 4b). In the absence of tBid, increasing autophagy capacity (AV levels) resulted in faster activation of mitophagy potential in the total mitochondrial population, as seen by the slopes (black lines) during the initial activation phase (gray shaded area), while the LIR activity of the three mutants reflected in the maximal mitophagy potential levels. Furthermore, an increase in AV levels reduced the time to phenotype commitment (dashed red line). Overall, these results indicate that increased autophagy capacity positively impacts mitophagy induction activity.

Subsequently, in order to understand the impact of autophagy capacity on minimizing mitochondrial population response to apoptosis signaling, we simulated Bnip3 pre-activation followed by tBid activation, at different AV levels distributed randomly (Fig. 4c). Bar graphs represent cellular responses at *t* = 100, with circles indicating individual cell response determined by the number of mitochondria “committed” to the mitophagy phenotype. Decreasing autophagic capacity by approximately 4-fold relative to standard (Fig. 4c, red box) suppressed the number of mitochondria with mitophagy phenotype by approximately 25 % and 13 % for Bnip3 WT and 2SE, respectively. On the other hand, increased AV content promoted mitophagy, with AV = 200 resulting in 2.2- and 1.7-fold increases for Bnip3 WT and 2SE, respectively (Fig. 4c, Additional file 9: Figure S9A). In contrast, the inactive Bnip3 LIR mutant 2SA showed no activity irrespective of AV level.

Notably, cytochrome *c* release was reduced by increasing autophagy capacity (Fig. 4c) further suggesting that enhanced pre-activation of mitophagy activity prior to tBid activation can bolster suppression of apoptosis. As expected, cytochrome *c* was released in a single burst (Fig. 4c, right lower panel), with total cytochrome *c* release (right upper panel) a result of the size and duration of the burst. The 2SA (dark blue) mutant exhibited a peak almost double of WT (light blue), with the release more prominent for a prolonged period of time. These results suggest that the timing of tBid activation plays a crucial role in regulating mitochondrial population phenotype response.

Hence, in order to explore the impact of timing, we delayed tBid expression to a time point still in the activation phase of mitochondrial decision process (*t* = 10), yet before phenotype commitment, hence still allowing for pathway competition (Fig. 4d). Albeit observing the same qualitative behavior of increasing mitophagy activity with increasing autophagy capacity, the level of mitochondrial commitment to mitophagy was significantly increased, and more importantly, apoptotic cytochrome *c* release capacity was further reduced by the later onset of tBid activation for all levels of AV compared to the earlier tBid activation time (Fig. 4c; see Additional file 9: Figure S9B for more scenarios). It should be noted, the behavior of WT and 2SE seem less distinct in both mitophagy and cytochrome *c* release responses, as if the impact of the constitutively active LIR domain is not as consequential. This observation is due to the delayed tBid activation giving mitochondria for both WT and 2SE more time to preferentially activate the mitophagy pathway (albeit the 2SE more strongly). Hence, by the time tBid is activated, both WT and 2SE are already far into committing to the mitophagy pathway, resulting in less competitive impact of tBid.

**Fig. 5:**
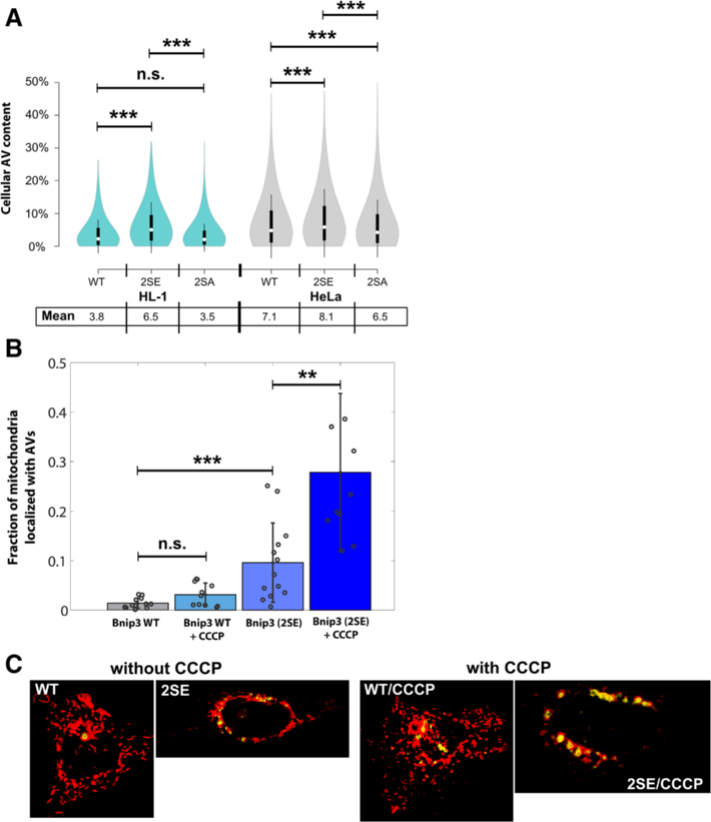
Validation of increased mitochondria sequestration with autophagic flux capacity. **a** HL-1 and HeLa cells stably expressing GFP-LC3 were transfected with RFP-Bnip3 WT, 2SA (inactive LIR) and 2SE (active LIR). Imagestream X measurements of cellular autophagosome content in HL-1 and HeLa cell lines were performed at 48 h of expression. The distribution of population measurements is shown as a Violin plot, with median indicated on plot, and mean response listed. **b** HeLa cells were transfected 24 h with GFP-LC3B and either RFP-Bnip3 WT or 2SE, and treated 6 h with CCCP (20 μM). From acquired Z-stacks, channels were segmented and the co-localization of mitochondria with autophagosomes was calculated on a slice-by-slice basis. The co-localized fraction is reported. **c** Examples of single-cell, Z-stack projections of mitochondria (red) with co-localized autophagosomes (yellow) for WT and 2SE mutant in MCF7 cells

Thus, the results predict that apoptosis signaling, under conditions of Bnip3-mediated mitophagy, is negatively regulated by autophagy capacity. To test this hypothesis, we first measured AV content levels in Bnip3-expressing HeLa and HL-1 cells (Fig. 5a, Additional file 10: Figure S10A). Compared to WT, 2SE AV levels were increased, and 2SA levels were decreased. In addition, in both cell types, overall, the AV fraction under Bnip3-mediated mitophagy conditions was slightly higher than steady-state AV measurements (Fig. 4a), but inferior to induction capacity, supporting model predictions that AV content or access limits mitophagy induction (Figs. 4b-4c), and hence apoptosis activation potential.

Furthermore, we measured within 3D stacks of single HeLa cells, the effect of autophagy induction on sequestration of Bnip3-targeted mitochondria, in order to confirm co-localization of Bnip3 mitochondria to autophagosomes (AVs). At 24 h of expression, the fraction of mitochondria sequestered by autophagosomes in 2SE mutant was about 7-fold higher than WT Bnip3 (Fig. 5b, Additional file 10: Figure S10B). Also, we previously demonstrated that CCCP treatment does not increase AV binding to activated LIR [14]. However, following CCCP (20 μm) treatments, which activates autophagy via AMPK [52], both WT and 2SE exhibited roughly 2 to 3-fold increases in sequestration respectively, illustrated in the images showing co-localization (yellow) of mitochondria (red) with autophagosomes (Fig. 5c).

Together, these results evidence that autophagosome content is a limiting factor during mitophagy, while increased autophagy promotes sequestration capacity, and suggest that cells with low autophagy capacity may be unable to mount a consequential mitophagy response.

### Autophagosome limitations can be overcome by mobility and increased dispersal

**Fig. 6:**
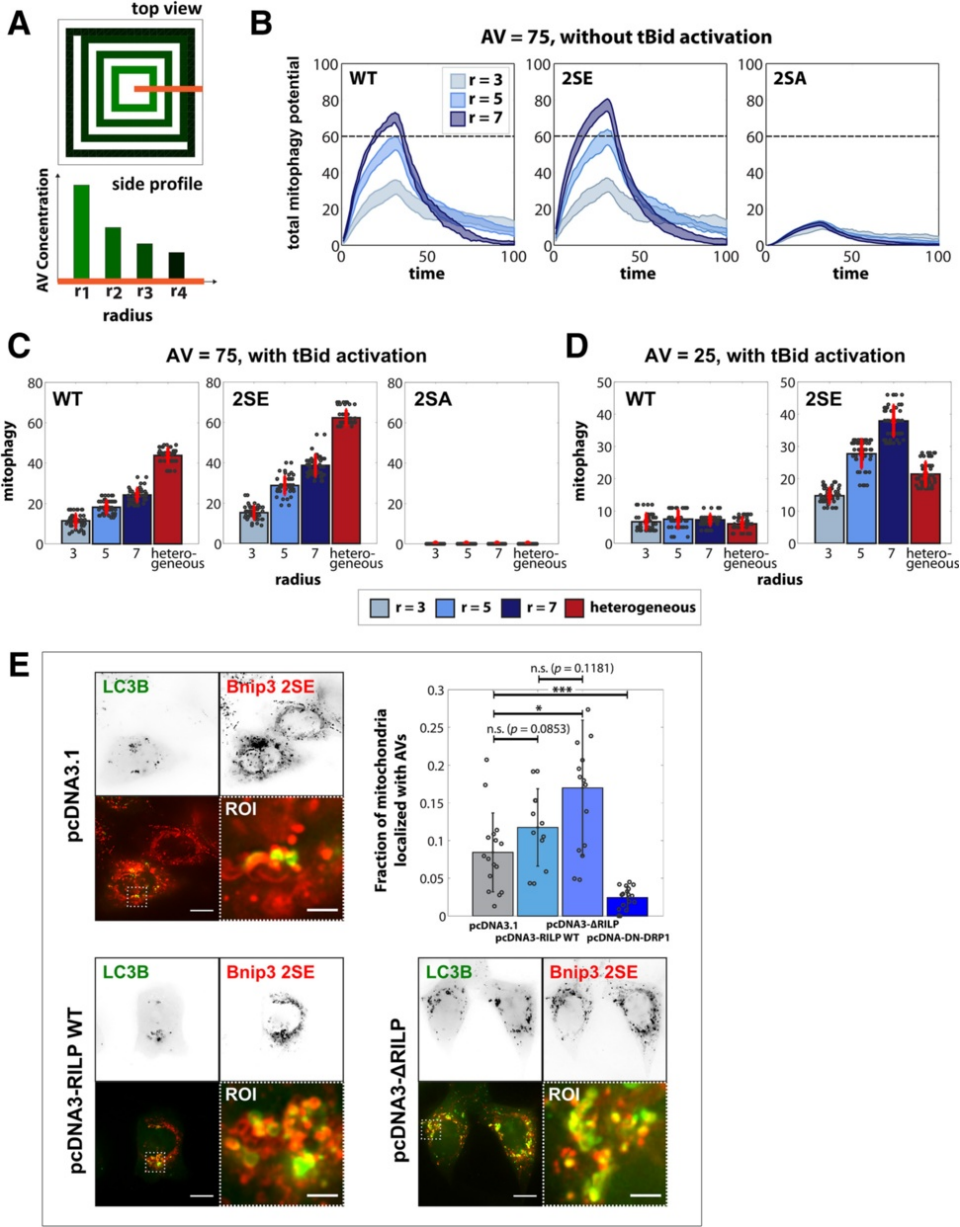
Impact of AV spatial localization on cell fate. **a** Schematic of AV distribution with (top view) increasing radii and (side profile) total AV level kept constant for all radii. **b** Total mitophagy potential of mitochondrial population in absence of tBid activation for low AV ( AV = 25) and increasing AV radial distribution (**c**) Number of mitochondria with mitophagy as final phenotype with increasing radii of AV distribution (blues) and tBid activation (at *t* = 5) at total AV = 75, compared to a heterogeneous AV distribution (red). **d** 3-fold decrease in AV level in comparison to **c** for Bnip3 WT and 2SE. **e** HeLa cells were co-transfected 24 h with GFP-LC3B and RFP-Bnip3 2SE, and either pcDNA3.1, pcDNA3-RILP or pcDNA3-ΔRILP. Representative images show GFP-LC3B (green) and RFP-Bnip3 2SE (red), with increased co-localization (yellow) for enforced peripheral localization of LC3B (pcDNA3-ΔRILP) compared to peri-nuclear (pcDNA3-RILP WT). From acquired Z-stacks, channels were segmented and the co-localization of mitochondria with autophagosomes was calculated on a slice-by-slice basis. Bar graph shows quantification of the fraction of sequestered mitochondria. Sample size for (**b**-**d**) was 50 runs each

Next, as an alternative perturbation strategy, we explored whether spatial distribution of autophagy machinery could be altered to influence mitophagy induction capacity. Specifically, we systematically concentrated AVs in increasing radial ring distributions, while keeping total AV level constant, to mimic localization of autophagic vesicles to the peri-nuclear region versus cellular periphery (Fig. 6a) and compared them to a random distribution.

In absence of tBid activation, increasing radii showed an increase in cellular mitophagy potential response for WT and 2SE (Fig. 6b). The simulations predict that maximizing AV distribution throughout the cell is more substantial to induce cellular mitophagy response, than localizing the same total AVs to one concentrated location.

Similarly, we repeated the analysis with tBid activation for both stationary (Additional file 11: Figure S11B) and mobile mitochondria (Fig. 6c) at increasing AV ring radii. At high AV levels (AV = 75), dynamic mitochondria were more efficient at inducing mitophagy than stationary, while increasing the radius increased mitophagic response further. By comparison, a heterogeneous distribution of AVs (red) increased mitophagy by approximately 50 % compared to the largest radius (dark blue) additionally illustrating the result that maximizing AV distribution optimizes mitophagy.

Interestingly, at low AV levels (AV = 25), Bnip3 WT exhibited no increase in mitophagic response for increasing radii, while the Bnip3 2SE mutant did, implicating the importance of LIR activity in inducing mitophagy sufficiently prior to apoptosis activation (Fig. 6d). This suggests that at low AV levels, mitophagy capacity can be increased by concentrating AVs to mitochondrial regions. We tested this hypothesis experimentally by altering the distribution of autophagy capacity in the cell by targeting Rab-interacting lysosomal protein (RILP), which tethers late endosomes and lysosomes to dynein-mediated retrograde microtubule trafficking (Fig. 6e) [53]. Remarkably, forcing concentration of endo-lysosomal activities to the cell periphery by expression of the pcDNA3-∆RILP mutant enhanced mitophagy response (yellow) for Bnip3 2SE by 45 % compared to a peri-nuclear localization induced by pcDNA3-RILP expression, and two-fold increase compared to control pcDNA3.1, supporting the model prediction (Fig. 6e, Additional file 11: Figure S11C).

Additionally, previously we showed experimentally that AV movement is restricted, while mitochondria are highly mobile (Additional file 5: Figure S5B). To explore the role of mobility of subcellular organelles, we investigated the impact of AV versus mitochondrial movement on cellular response behavior for all three Bnip3 mutants (Additional file 12: Figure S12). The results demonstrate that dual immobility was the least efficient with mitophagy activity determined solely by co-localization resulting from the initial random distribution of both species. Introducing movement to either organelle increased mitophagy activity by 50 % for WT and 2-fold for 2SE. Notably, enforcing mobility of both organelles simultaneously only increased cellular mitophagy response by a further 10 %, indicating that movement of a single species of organelle is sufficient to maximize interaction of mitochondria with autophagosomes.

Together, these results suggest that subcellular organelle mobility plays a crucial role in mitochondrial decision processes. However, there are limits to the contribution of movement to mitophagy activity, and localization of the autophagy machinery to the vicinity of mitochondria is most essential for efficient mitophagy.

### Subcellular distributions of Bcl2 signaling components underlie mitophagy regulation

Thus far, we demonstrated how spatial and dynamic variability of subcellular components contribute to shape the control of apoptosis signaling. Notably, even when we compensated for the lack of AVs by maximizing cell area coverage by autophagosomes, and ensured the mitophagy receptor was constitutively active by using the 2SE mutant, the mitophagy response was nevertheless not complete within the cell (Fig. 5b). Importantly, recent findings have demonstrated that fragmented mitochondria exhibit substantial heterogeneity in pro-apoptotic Bax/Bak protein levels, which is essential for MOMP and cytochrome *c* release [38, 39]. Functionally, heterogeneity can result in sub-populations of mitochondria resistant to MOMP. Evidence suggests that MOMP is not an all-or-nothing event. A small sub-population of mitochondria resisting MOMP activation can re-populate in a cell and help its survival [38].

**Fig. 7:**
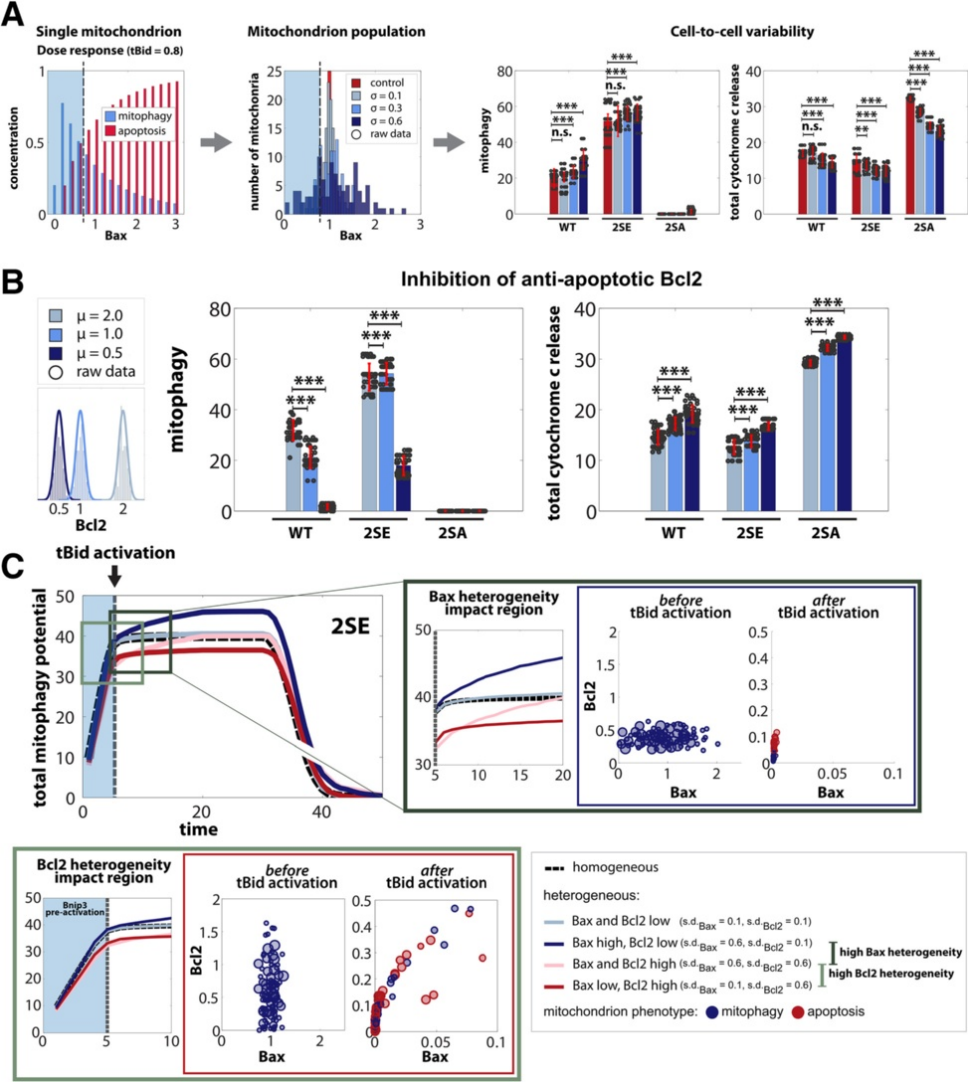
Impact of mitochondrial heterogeneity on cell fate. Systematic analysis of heterogeneity in Bax levels for mitochondrial population shows emergence of sub-populations. **a** (left) Single mitochondrion dose response to increasing tBid activation with (blue shaded) sub-population resistant to apoptosis stimuli. (middle) Histogram of mitochondrial population’s Bax level after randomly seeding each mitochondrion with Bax levels from Gaussian distributions (mean = 1, standard deviation = (0.1, 0.3, 0.6)), while keeping Bcl2 mean fixed (at 1). (blue shaded) emergence of small sub-population with very low Bax levels (right) Total number of mitochondria with mitophagy as final phenotype and total cytochrome *c* release for increasing Bax heterogeneity (**b**) Simulation of Bcl2 inhibition: Decreasing mean of anti-apoptotic Bcl2 (with s.d. = 0.1) while keeping the mean of Bax fixed (at 1) (**c**) Implementation of different combinations of heterogeneity in Bax and Bcl2 levels (see legend) compared to control (black line) at different stages of tBid activation (light green box is pre-tBid activation, dark green box is post-tBid activation). For all simulations sample size was 50 runs

Therefore, we implemented Bcl2 and Bax heterogeneity in the mitochondrial population to investigate the impact of each on the population, and consequently, on cellular behavior. As such, the mitochondria were randomly seeded with Bax and Bcl2 levels systematically taken from a Gaussian distribution with certain mean and standard deviation (Fig. 7a, ‘Mitochondrion population’). An increasing standard deviation represented Bax or Bcl2 increasing heterogeneity.

First, we investigated the influence of increasing heterogeneity in Bax by increasing the standard deviation of the Gaussian distribution, while setting initial Bcl2 values for all mitochondria equal to 1. Compared to control, where an homogenous mitochondrial population had initial Bax levels set to 1, increasing heterogeneity significantly increased the average number of mitochondria committing to the mitophagy phenotype in a cell (Fig. 7a, ‘Cell-to-cell variability’; Additional file 13: Figure S13A). This increase in decision in favor of mitophagy with increasing heterogeneity is due to the emergence of a sub-population of mitochondria with very low levels of Bax susceptible to the initial pre-activation of the mitophagy pathway yet resistant to subsequent tBid activation. This is evident in the dose response curve for a single mitochondrion post tBid activation (Fig. 7a, ‘Single mitochondrion’). The blue shaded area indicates the Bax levels at which mitophagy dominates despite tBid activation. Hence, with increasing heterogeneity the number of mitochondria with Bax levels in this regime increases (‘Mitochondrion population’, shaded region), contributing towards the increase in mitophagy activity in a cell, while reducing cytochrome *c* levels indicative of diminished MOMP activity.

Next, to explore the impact of inhibition of anti-apoptotic Bcl2, the overall mean of Bcl2 was decreased, while keeping the mean of Bax fixed. As expected, this lead to a decrease in mitochondrial population commitment to mitophagy phenotype, and increase in total cytochrome *c* release, due to decreased Bcl2 binding with Bax to inhibit apoptosis activation (Fig. 7b; Additional file 13: Figure S13B), consistent with experimental observation [38].

Finally, we investigated whether Bax and Bcl2 heterogeneities acted during different phases of the mitochondrial decision process. During Bnip3 pre-activation (Fig. 7c, blue shaded region), high Bcl2 heterogeneity (light green box, red lines) was able to reduce the activation rate compared to a control homogeneous population (dashed black), due to a small sub-population of mitochondria with low Bcl2 levels evading mitophagy pre-activation. On the other hand, high Bax heterogeneity (dark blue) showed no deviation from control. However, post tBid activation (dark green box) high heterogeneity in Bax (dark blue and light red) caused an increase in mitophagic response due to the aforementioned emergence of a small sub-population with low Bax (Fig. 7a).

Hence, these results suggest that Bax and Bcl2 heterogeneities impact mitochondrial (and hence cellular) activity at different stages of signaling adaptation, and the emergence of sub-populations help explain the observed extensive cell-to-cell variability even under conditions of constitutively-active LIR and high AV content.

## Conclusion

In this manuscript, we developed a multi-scale model, using ODEs to simulate individual mitochondrial dynamics, and rules-based decisions to simulate mitochondrial population state behavior in space and time. We focused our model on Bnip3, which is a sensitizer to BH3-only protein [40], an inducer [46] and sensor of ROS [43], and crucially, contains a phosphorylation-regulated LIR domain to signal mitophagy prior to MOMP [14].

### Model-based insights into the Bnip3 mode of mitophagy

**Fig. 8:**
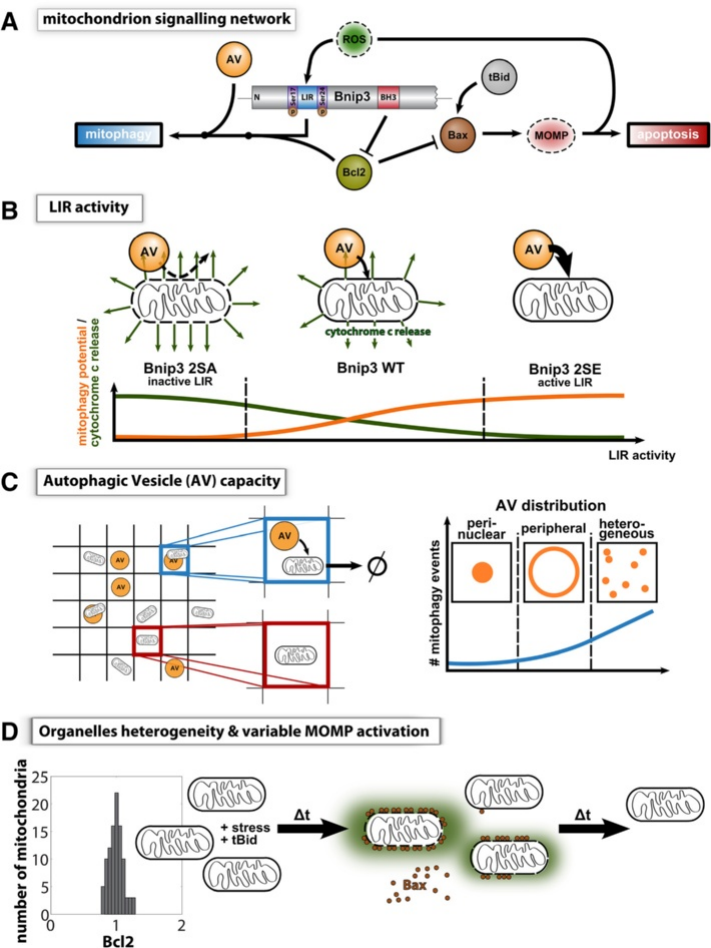
Illustration of key findings. **a** Bnip3 dual-functionality and crosstalk between mitophagy and apoptosis pathways. **b** Bnip3 LIR activity determines interaction with LC3, and influences MOMP activation (and consequent cytochrome *c* release) (**c**) Level of mitophagy in a cell is dependent on mitochondrial autophagy potential (AV content), and spatial localization of autophagic vesicles (**d**) Subcellular heterogeneity at mitochondria, specifically proteins at crosstalk between both pathways, impact individual mitochondria response to stress and apoptosis signaling, resulting in emergence of sub-populations able to influence cell fate

From our ODE model (Fig. 8a; Additional file 1: Figure S1) we can show how Bnip3 dual functionality shifts between BH3-mediated inhibition of Bcl2, thereby increasing activation of Bax by tBid, and LIR-mediated mitophagy, which reduces apoptotic signaling (Fig. 8b). Our model also proposes a simple explanation for how increased Bcl2/x_L_ activity impacts apoptosis signaling by enhancing mitophagy: coincidently Bcl2/x_L_ slows apoptosis induction, which reduces Bax mediated suppression of autophagy [27], and promotes mitophagy in the absence of apoptotic stimuli (Additional file 3: Figure S3) [14]. Several lines of evidence suggest that mitophagy-sensitized conditions occur under physiological and pathophysiological conditions. *In vivo*, Bcl-x_L_ and Bnip3 expression is positively (and negatively) correlated in several disease and non-disease states (Additional file 14: Figure S14). Moreover, the autophagy receptor Bnip3L/Nix, a close homologue of Bnip3 [13], and Bcl-x_L_ are co-upregulated during red blood cell (RBC) maturation [54]. We suggest that co-increase of Bnip3/Bnip3L and Bcl2/x_L_ can alter the mitochondrial apoptotic pathway, providing autophagy capacity is sufficiently high. In addition, our modeling offers simple explanations for the impact of ROS through a positive feedback loop: Bnip3 requires ROS activation [43], and Bax activation generates ROS [30, 33–35]. Although this feedback is simplified, and discounts non-apoptotic ROS amplification [33, 34], an environmental decrease of ROS levels suppressed mitophagy induction (Additional file 7: Figure S7A-S7C), which is consistent with recent *in vivo* evidence that ROS suppression in the heart can impede mitophagy [32]. However, ROS signaling integrates with autophagy induction [31], alternative mitophagy modes [55], lysosomal death signaling [56] and apoptosis [30], and therefore further work is required to better elucidate its role in regulating additional pathway crosstalk.

It is important to note that in this study we uniquely addressed the role of Bnip3-mediated mitophagy, and additional mitophagy programs occur in mammalian cells [9], which undergo distinct crosstalk with apoptosis signaling. FUNDC1 is a phosphorylation-regulated mitophagy receptor, which is activated during hypoxia conditions [23, 57]. Interestingly, while FUNDC1 knockdown was shown to have no impact on apoptosis [23], it was recently shown that FUNDC1 activity can be negatively regulated by Bcl-x_L_ [58], suggesting that it could be activated by Bnip3-antagonism of Bcl-x_L_, or in subpopulations of Bcl-x_L_-poor mitochondria. Further, we note clear distinctions between Bnip3 and PINK1/Parkin modes of mitophagy. While PINK1 activation of Parkin is promoted by mitochondrial membrane depolarization [24], Bnip3 targets mitochondria in the polarized state to autophagosomes [14]. In addition, differences in mitochondrial mobility occur between the pathways. PINK1 targets Miro, rendering mitochondria static prior to clearance [59], while we report that mitochondria targeted by active Bnip3-2SE are highly mobile (Additional file 5: Figure S5B). Finally, while Bnip3 and Parkin mitophagy modes have distinct roles in basal mitochondrial quality control *in vivo* [60], the relationship between the pathways under apoptotic conditions is undetermined, and expected to be complex due to extensive crosstalk between Parkin and Bcl2 signaling. For example, pro-survival Bcl2 members suppress mitophagy through inhibition of Parkin translocation to depolarized mitochondria, while BH3-only proteins promote Parkin translocation and induction of mitophagy under conditions of executioner caspase inhibition [61]. In addition, Parkin can differentially impact MOMP activation, promoting MOMP via degradation of Mcl-1 [62], or suppressing Bax activity via direct ubiquitylation [63]. As our model gives valuable insight into the dynamics of Bnip3 subcellular signaling, we propose that our approach will be useful to explore the emergence of possible behaviors arising from crosstalk with the Parkin machinery and other mitophagy programs.

### Subcellular autophagosome capacity and localization restricts mitochondrial sequestration

Recent findings suggest that autophagosome production can be directed to depolarized mitochondria [64] and consistent with this finding we have observed that autophagosomes accumulate at mitochondria targeted with LIR-inactive mutant Bnip3, without binding to mitochondria, indicating that local AV production can also occur during Bnip3-mediated mitophagy [65]. However, based on our evidence that autophagosome content is limiting sequestration (Figs. 4a-c) and that autophagosome mobility is also limited (Additional file 5: Figure S5B), we explored the impact of spatial localization of this limited capacity on sequestration events. Strikingly, our multi-scale model predicts that AV levels and their spatial localization help determine the extent of mitophagy induction (Fig. 8c). While organelle mobility enhanced mitophagy activity, it was the level and spatial distribution of AVs that determined mitophagy activity (Figs. 4, 5, 6). From a biological point of view, this emergent behavior was surprising, as Bnip3 activates autophagy via BH3 interactions [66], and Bnip3 binding to Rheb also suppresses autophagy inhibition by mTOR [67]. However, quantitative analysis of autophagy and mitophagy suggests that AV up-regulation by Bnip3 is not maximal compared to the activation of autophagy by mTOR inhibition (Figs. 4a and 5a), and we show that by inducing autophagy with CCCP in HeLa cells, sequestration for both WT and 2SE Bnip3-targeted mitochondria was significantly increased (Fig. 5b). These findings support model predictions that autophagosome content is a limiting factor during mitophagy. Furthermore, we found *in silico* that increasing AV spatial distribution can promote mitophagy response, under conditions of limited AV content. This was tested experimentally by altering the spatial distribution of endo-lysosomes. We report that localization of autophagic machinery to the cell periphery, as opposed to the peri-nuclear region, increased the fraction of sequestered mitochondria (Fig. 6e). Thus, mitophagy activation prior to apoptosis induction is required for a pro-survival function, with increasing mitophagy levels showing more effective apoptosis suppression (Fig. 4c-4d and Additional file 9: Figure S9B). In addition, autophagy capacity, which varies between cell type and conditions (Fig. 4a), determines whether cells can mount a consequential mitophagy response. As such, autophagosome production capacity represents a target for regulating mitochondrial-amplification of apoptosis.

Overall, the above predictions describe how the role of environmental conditions, oxidative stress handling and autophagy induction capacity contribute to mitochondrial population heterogeneities. However, remarkably, even with enhanced induction of mitophagy (Figs. 5 and 6e), sequestration of the mitochondrial population was never maximal, indicating that additional factors determine the targeting of mitochondria to autophagosomes.

### Bcl2 member distributions among mitochondrial population regulate subcellular mitophagy engagement

Bcl2 and Bax heterogeneities in populations of mitochondria were recently described to result in variable MOMP activities [38, 39]. Simulating increasing Bax heterogeneity, while maintaining mean Bax levels of the population, resulted in sub-populations of mitochondria with very low levels of Bax, resistant to tBid activation, and hence, resistant to apoptosis signaling (Fig. 8d), consistent with experimental results [38]. This small sub-population increased the number of surviving mitochondria in a cell compared to control (Fig. 7a), which suggests a mechanism to establish a minimum number of surviving mitochondria which could re-populate the mitochondrial population if cytochrome *c* [68] or Smac [69] is degraded prior to lethal executioner caspase activation. Notably, additional sources of heterogeneity were discounted here, and further work will be required to determine their role in post-MOMP signaling [37, 70]. Overall the ensemble of simulation and experimental findings suggests that Bnip3-mediated mitophagy could be functionally impacting sub-lethal, oncogenic MOMP activity [7], and warrants further investigation in the role of mitophagy in cellular resistance and recovery from executioner caspase activation [4–6].

### Multi-scale modeling approach to capture impact of subcellular heterogeneities and mechanisms on emergence of cellular behavior

Here, the hybrid nature of our model permits insights into the dynamics of defined pathway topologies from BH3 and LIR domain-specific behaviors, while agent-based modeling readily incorporates biologically-relevant sources of information on multiple length scales. By following the evolution of each autonomous agent (here mitochondria), predictions can be made about the temporal emergence of global behavior from local interactions and any possible amplification effects of subcellular heterogeneities on cellular fate. Thus, our model presents one solution of how to investigate multi-functionality of apoptosis and autophagy members [71], operating on different time scales. Moreover, while our model is highly simplified in terms of parameters, by integrating topological and spatial dynamics, we are able to computationally test scenarios, and directly compare simulation findings to experimental results. As such, we propose that our approach reduces dependency on cell-specific parameters, and even more importantly, experimental bias, while facilitating integration of qualitative and quantitative pathway information, which compose the vast majority of biological reports. However, we also suggest that the scalability of agent-based modeling will permit the inclusion of mechanisms required for quantitative understanding, for example spatial and temporal regulation of AV formation and degradation [65].

In conclusion, our model reveals that heterogeneous, localized signals drive the behavior of each mitochondrion, and the collective contributions can regulate cellular apoptosis fate. Interestingly, the emergence of small sub-populations leaves the possibility of steering cell fate by manipulating sub-populations through spatial directionality or seeding organelles with a wanted level of heterogeneity. Such understanding of how subcellular heterogeneity impacts cell survival is of clinical importance, as this offers novel insight in to approaches to either kill, or conversely improve cell survival. Overall, we propose our modeling approach as an excellent strategy to integrate high content, quantitative and qualitative behavioral and mechanistic findings and formulate testable hypotheses.

## Materials and methods

### Biochemical modeling of mitochondria

The ODE model for each mitochondrion’s mitophagy versus apoptosis pathway decision was built and run in MATLAB R2011b using solvers in the Systems Biology Toolbox 2 (SBTOOLBOX2). A topological approach was taken for the signaling network analysis rather than using parameter estimates, in order to emphasize the importance of signaling interaction dynamics. Furthermore, given the observation-based qualitative nature of the ODE parameter values, an exact conversion to real-time scale was not of relevance, but rather the qualitative behavioral response. Forward reaction rates were assumed to be faster than reverse reaction rates, with *r*_*f*_ = 0.1 and *r*_*r*_ = 0.01. The Bnip3-AV binding rates for the various Bnip3 mutants were taken to be *r*_*f*_^*WT*^ = 0.05, *r*_*f*_^2*SE*^ = 3, *r*_*f*_^2*SA*^ = 0.001. As initial levels, intrinsic cytochrome *c* and caspase 3 were set to 1, while all other levels were initialized to zero. Bnip3 was also set to 1, unless pre-activated, for which inactive Bnip3 was at 80 % and active Bnip3 at 20 %. Bax and Bcl2 levels were randomly initialized from a Gaussian distribution depending on perturbation type of interest. BH3 and AV levels were seeded from the local environment.

### Integration of mitochondrion population with heterogeneous environment in an agent-based model

The agent-based model was built and run in MATLAB R2011b, and typically, all conditions were run for a sample size of 50 simulations, each with 200 time steps. A population of 100 mitochondria, each with individually tracked ODE level values, was randomly distributed on a 20 × 20 grid. Each grid box was assumed to be approximately the size of a mitochondrion (1 μm × 1 μm), and the total system size about the size of a single cell (20 μm × 20 μm). Mitochondrial movement was random and discrete, jumping to a nearest neighbor with every time step. Since, mitophagy requires mitochondrial fragmentation [45, 72] there were no fission/fusion events. The mitochondrion-environment interaction was restricted to its current position, with local levels (ROS, BH3, AV) used as initial conditions for every ODE update time step. All other levels were stored and used for the next ODE update time step. Detailed rules for mitochondrial state changes are given in Additional file 2: Figure S2.

Three environmental layers were implemented: ROS, AV, tBid. AVs were generally randomly distributed and kept stationary, except for comparison with dynamic AVs (Additional file 12: Figure S12) and radial distribution (Additional file 11: Figure S11B). ROS and tBid were continuous values in each box with diffusion between nearest neighbors. There were two sources of ROS production: general non-mitochondrial with constant rate [48], and Bax-mediated mitochondrial. A general ROS degradation by superoxide anions (SOD) was assumed as they have the highest enzyme activity. Lastly, for simulations with tBid, activation occurred at *t* = 5 (except Fig. 4d), at a constant rate of 0.25 per time step for 4 time steps, and a level of [tBid]_model_ = 0.9. Similar to our experiments, we chose a tBid dose that would activate apoptosis but not overpower mitophagy.

Note, due to the two time scales in the model, the ODE updates were run every second agent-based model time step. Based on simulation observations a decision period of 15 time steps was chosen for mitochondria to ‘lock-in’ to a phenotype, which allows sufficient time to adapt to competing signals and/or any perturbations to their neighborhood.

### Cell lines and treatment

Bax/Bak double-knock out mouse embryonic fibroblasts (ATCC® CRL-2913™) were maintained in IMDM medium containing L-glutamine and HEPES and supplemented with 10 % FBS, non-essential amino acids and penicillin/streptomycin/amphotericin B. Human MCF-7 (Cell Lines Services, Heidelberg, Germany), Panc-1, Capan1 (obtained from the Department of General Surgery, University of Heidelberg, Germany), and HeLa Kyoto cancer cell lines were maintained in full medium, consisting of DMEM, 10 % FBS, L-glutamine, non-essential amino acids, penicillin, streptomycin, amphotericin B. The HL-1 cardiac myocyte cell line was maintained in Claycomb medium supplemented with 10 % FBS, 0.1 mM norepinephrine, 2 mM L-glutamine, penicillin/streptomycin/amphotericin B. The human pancreatic duct epithelial HPDE cell line was maintained in KGM medium supplemented with bovine pituitary extract, hEGF, insulin, hydrocortisone, gentamicin and amphotericin B (Lonza). MCF10A human breast epithelial cells were cultured in DMEM/F12 medium supplemented with 5 % horse serum, 20 ng/ml epithelial growth factor, 0.5 g/ml hydrocortisone, 10 μg/ml insulin, 100 units/ml penicillin, 100 units/ml streptomycin, and 0.25 μg/ml amphotericin B.

Expression vectors used in the study were previously described [11]. Transient transfections were performed using JetPRIME (Polyplus) transfection reagents. MCF-7 cell lines stably expressing fusion proteins were generated via selection with 1 mg/ml G418. To generate all other stable cell lines, pWIPI lentiviral vectors containing GFP-LC3B were generated in 293 T cells. Cells were infected using lentivirus-containing 293 T cell supernatant.

Treatments with CCCP (20 μM), RAD001 (100 nM) and Bafilomycin A1 (BafA1, 100 nM) were performed in fully supplemented cell culture medium (FM), or in glucose-containing Krebs Henseliet Solution (Sigma) for nutrient deprivation (ND) conditions.

### Fluorescence imaging and image analysis

A DeltaVision RT microscope system (Applied Precision) equipped with a × 60 oil immersion objective was employed for widefield fluorescence microscopy. Cells were either fixed with 4 % paraformaldehyde, or imaged live for time-lapse imaging at 37C in a humidified chamber, with 5 % CO2. Images of representative cells were captured using the Z-axis scan function, or, when indicated, as Z-stacks with 0.3 μm step sizes. Acquired images were deconvolved (Softworx, Applied Precision). Image analysis and preparation was performed using ImageJ (rsbweb.nih.gov/ij/). Representative images shown are total intensity projections (Z-axis scans) or maximal intensity projections (Z-stacks). Inverted grey-scale was chosen for displaying single color channels for optimal detail visibility. In merged color images, pseudo-colors correspond to font colors of protein labels within single color images.

### Quantifications of mitophagy from fluorescence microscopy data

HeLa cells were plated in 8-well microscopy μ-slides (iBidi), and as indicated, were transfected with combinations of GFP-LC3 and Bnip3 WT or 2SE-RFP, and pcDNA3.1, pcDNA-DN-Drp1, pcDNA3-RILP, or pcDNA3-ΔRILP, and at 24 h of expression subjected to the indicated treatments. Cells were then fixed with paraformaldehyde (PFA) (4 % PFA in PBS, pH 7.4). For immunostaining, cells were permeabilized with 0.3 % Triton X-100 in PBS, and blocked with 3 % BSA. Following, cells were incubated with primary antibodies against Tom20 (Santa Cruz; no. sc-11415 ), and at room temperature for 1 h. Fluorescent staining was performed for 30 min at room temperature using highly cross-absorbed Alexa Fluor 647 secondary antibodies (Life Technologies).

From Z-stacks, single cells were cropped for analysis by ImageJ. Binary masks for each slice within ≥ 10 representative Z-stacks per condition of (i) RFP-Bnip3 or Tom20-labeled mitochondria and (ii) GFP-LC3 were generated by image segmentation. Slice-by-slice co-localization of mitochondrial and AV masks were calculated using the Boolean AND function. All slices for each binary stack were summed, and the ratio of area calculated from mitochondria-localized AV over total mitochondria content is reported as a cellular fraction (range 0–1). Measurements were obtained from at least 3 independent experiments.

### Image-based flow cytometry

GFP-LC3-expressing cell lines were seeded into each 6-well or 12-well plates and submitted to indicated experimental conditions. Drug treatments were performed at 24 h following plating for a duration of 3 h. Bnip3 expression experiments were analyzed at 48 h post-transfection. Following treatments, cells were trypsinized, fixed in 4 % paraformaldehyde (PFA) for 20 min, resuspended in PBS and analyzed using an ImageStream X system (Amnis, Seattle, WA). For image analysis, IDEAS software (Amnis) was used. Briefly, the population of in-focus, single cells was selected for analysis. Segmentation masks of single cells and intra-cellular GFP-LC3 autophagy vesicles (AVs) were generated. From these masks, cellular intensity fractions for AVs were calculated for each cell. Cell population measurements are represented as boxplots, with mean and/or medians indicated. In Fig. 4a, autophagy flux is reported based on fold change between steady-state and cumulative conditions (mean cumulative (+ Baf ) AV fraction/mean steady-state AV fraction). In Fig. 5, mean population responses for steady-state AV fractions of WT and mutant RFP-Bnip3 transfected cells are reported. Population measurements are representative of at least 3 independent experiments.

### Statistical analysis

The probability of statistically significant increases or decreases between conditions was determined using the two-sample Kolmogorov-Smirnov test indicated in the figures. Statistical significances in the figures are: n.s. = *p* > 0.05, * = *p* ≤ 0.05, ** = *p* ≤ 0.01, *** = *p* ≤ 0.001. Values are expressed for bar graphs as mean ± s.d. as well as individual data points included as scatter points. All other statistics are presented in the relevant Supplementary Figures.

## Additional files

Additional file 1: Figure S1. Equations for ODE model of a single mitochondrion. (PDF 139 kb) Additional file 2: Figure S2. Flow diagram of time iteration loops for implementation of all update events in the multi-scale model. (Dark blue) Agent-based model updates (light blue) ODE updates for all mitochondria (dashed dark green) ODE update rules (dashed light green) mitochondrial state definitions (dashed gray) parameter values for model. (PDF 928 kb) Additional file 3: Figure S3. Experimental validation of two distinct pathways via Bnip3. Bax/Bak DKO cells were co-transfected 24 h with GFP-LC3B and either mito-RFP or RFP-Bnip3 2SE. Bax/Bak DKO cells with apoptosis disabled show no co-localization of autophagosomes (GFP-LC3) with mitochondria (Mito-RFP). Cells expressing active-LIR RFP-Bnip3 2SE mutant show co-localization. (PDF 1788 kb) Additional file 4: Figure S4. Mitophagy (blue) versus apoptosis (red) activity for different combinations of Bnip3 and Bcl2 levels, as a function of increasing tBid activation (direction of arrow) and 20 % Bnip3 pre-activation. Experimentally observed dual-functionality of Bnip3 only qualitatively reproducible in regime with Bnip3 and Bcl2 simultaneously at high levels; otherwise apoptosis pathway dominates. (PDF 486 kb) Additional file 5: Figure S5.
**A** Experimental validation of fragmented mitochondria in stressed cells, underlying model assumption of no mitochondrial fission/fusion events. HeLa cells were co-transfected 24 h with indicated combinations of mito-GFP, GFP-Bnip3, and RFP-Bcl-xL. (Fused mitochondria) Individual expression of Bcl-x_L_ (top) or Bnip3 (middle) show elongated mitochondrial structure, (Fragmented mitochondria) Co-expression of Bcl-x_L_ and Bnip3 exhibits highly fragmented mitochondria and co-localization (yellow). **B** HeLa cells were co-transfected 24 h with GFP-GATE16 and RFP-Bnip3 2SE. Projections of time lapse images for autophagosomes (Gate 16) and mitochondria (Bnip3) depict organelle mobility. After treatment (see *Methods*), images were taken in 30 s increments for 30 min, and overlaid. The white regions for autophagosomes indicate immobility, while the wide dispersal for mitochondria is indicative of high mobility. (PDF 3157 kb) Additional file 6: Figure S6.
**A** Comparison of heterogeneous versus homogeneous AV distribution in a homogeneous mitochondrial population with Bnip3 pre-activation. Scatter points track mitochondrial content individually, with size and color depth indicating level of mitophagy (blue) or apoptosis (red) potential at every time step. Total mitophagic content in mitochondrial population (blue line) indicates the population response. The population exhibits three phases (insets): activation of signaling pathways (light gray), competition to commit to a phenotype (dark gray), and a committed phase during which phenotype is executed (olive). Runs show cell-to-cell variability (blue shaded area) **B** tBid activation induces some variability due to emergence of heterogeneous ROS environment. Total mitophagic and apoptosis potential curves were averaged of 50 sample runs. (PDF 239 kb) Additional file 7: Figure S7. ROS degradation versus production comparison. Keeping ROS degradation rate constant, variation in non-mitochondrial ROS production rates in Bnip3 WT with **A** 5-fold decrease compared to control in **B** and **C** 4-fold increase. Results show mitochondrial mitophagy decisions (blue histograms) and consequent apoptosis activity indicated by cytochrome *c* release (red). Simulations of varying ROS degradation rates show little impact on **D** mitophagy (blue histograms) and **E** cytochrome *c* release. Sample size was 50 runs for each condition. Tables show statistics (at *t* = 100). (PDF 281 kb) Additional file 8: Figure S8. Statistics for Fig. 4a. (PDF 338 kb) Additional file 9: Figure S9.
**A** Statistics for Fig. 4c **B** Impact of different combinations of AV level versus tBid activation time on cytochrome *c* release. Sample size was 50 runs for each condition. (PDF 194 kb) Additional file 10: Figure S10. Statistics for Fig. 5. (PDF 53 kb) Additional file 11: Figure S11.
**A** Simulations of reducing AV levels three-fold compared to standard levels (AV = 75, Fig. 6b) and no tBid activation. **B** Comparison of stationary mitochondria (solid) versus dynamic mitochondria (hashed) for increasing radii of AV distribution rings with tBid activation (at AV = 75) compared to heterogeneous AV distribution (red). Sample size was 50 runs for each condition. **C** Statistics for Fig. 6e. (PDF 477 kb) Additional file 12: Figure S12. Comparison of mitochondrial versus AV mobility: movement of either subcellular organelles (middle two groups) compared to immobile system (left group), and mobility of both species (right group), with corresponding total cytochrome *c* release behavior. Table shows statistics for each condition with sample size of 50 runs each. (PDF 483 kb) Additional file 13: Figure S13. Statistics for Figs. 7a and 7b. (PDF 45 kb) Additional file 14: Figure S14. Dataset of several disease and non-disease states show positive correlation of Bcl-x_L_ and Bnip3 expression. Using the R2: Genomics Analysis and Visualization Platform (http://hgserver1.amc.nl/cgi-bin/r2/main.cgi), a 2D gene overview was performed across all datasets, which include mRNA expression from diseased and normal tissues. The R coefficients for datasets 133a and u133p2 are reported. (PDF 122 kb)

## Abbreviations

Bnip3: Bcl2/adenovirus E1B 19 kDa protein-interacting protein 3
tBid: truncated Bid
CCCP: Carbonyl cyanide *m*-chlorophenyl hydrazone
ODE: Ordinary differential equation
ABM: Agent-based model
ND: Nutrient deprivation
FM: Full medium
Baf: Bafilomycin
RILP: Rab-interacting lysosomal protein
