# Supplementary material for: Autophagy capacity and sub-mitochondrial heterogeneity shape Bnip3-induced mitophagy regulation of apoptosis
Source: Cell Commun Signal. 2015 Aug 8;13:37. doi: 10.1186/s12964-015-0115-9 (PMC4528699; doi:10.1186/s12964-015-0115-9)
Supplement: Additional file 1: Figure S1. — Equations for ODE model of a single mitochondrion. (PDF 139 kb) [file 12964_2015_115_MOESM1_ESM.pdf]

Supplementary Figure S1

Reaction Rates

$$f([Bnip3]_{activation}) = k_f^{Bnip3Activation}[Bnip3_{inactive}][ROS]$$

$$f([Bnip3{:}LC3]_{binding}) = k_f^{Bnip3LC3binding}[Bnip3{:}LIR][LC3] - k_r^{Bnip3LC3binding}[Bnip3{:}LIR]$$

$$f([BH3{:}Bcl2]_{binding}) = k_f^{BH3Bcl2binding}[BH3][Bcl2] - k_r^{BH3Bcl2binding}[BH3{:}Bcl2])$$

$$f([Mitophagy]_{activation}) = k_f^{MitophagyActivation}[Bnip3{:}LC3][CytoC_m]$$

$$f([MOMP{:}ROS{:}CytoC]_{release}) = k_f^{MOMP:ROS:CytoCrelease}[BH3{:}Bax][CytoC_m]$$

$$f([mitochondrialApoptosis]\quad) = k_f^{MitochondrialApoptosis}[Caspase3][CytoC_r]$$

$$f([BH3{:}Bax]_{activation}) = k_f^{BH3BaxActivation}[BH3][Bax]$$

$$f([Bcl2Bnip3]_{binding}) = k_f^{Bcl2Bnip3Binding}[Bcl2][Bnip3_{active}] - k_r^{Bcl2Bnip3Binding}[Bnip3{:}Bcl2]$$

$$f([LIR]_{phosphorylation}) = k_f^{LIRPhosporylation}[Bnip3{:}Bcl2] - k_r^{LIRPhosphorylation}[Bnip3{:}LIR]$$

Differential Equations

$$\frac{d[ROS]}{dt} = [Bnip3]_{activation} + [MOMP{:}ROS{:}CytoC]_{release}$$

$$\frac{d[Bnip3{:}Bcl2]}{dt} = [Bcl2Bnip3]_{binding} - [LIR]_{phosphorylation}$$

$$\frac{d[Bnip3{:}LC3]}{dt} = [Bnip3{:}LC3]_{binding} - [Mitophagy]_{activation}$$

$$\frac{d[Bcl2]}{dt} = -[BH3{:}Bcl2]_{binding} - [Bcl2Bnip3]_{binding}$$

$$\frac{d[Bnip3_{active}]}{dt} = [Bnip3]_{activation} - [Bcl2Bnip3]_{binding}$$

$$\frac{d[BH3{:}Bax]}{dt} = -[MOMP{:}ROS{:}CytoC]_{release} + [BH3{:}Bax]_{activation}$$

$$\frac{d[CytoC_r]}{dt} = [MOMP{:}ROS{:}CytoC]_{release} - [mitochondrialApoptosis]$$

$$\frac{d[Bnip3{:}LIR]}{dt} = -[Bnip3{:}LC3]_{binding} + [LIR]_{phosphorylation}$$

$$\frac{d[CytoC_m]}{dt} = -[Mitophagy]_{activation} - [MOMP{:}ROS{:}CytoC]_{release}$$

$$\frac{d[BH3{:}Bcl2]}{dt} = [BH3{:}Bcl2]_{binding}$$

$$\frac{d[BH3]}{dt} = -[BH3{:}Bcl2]_{binding}$$

$$\frac{d[Bnip3_{inactive}]}{dt} = -[Bnip3]_{activation}$$

$$\frac{d[Bax]}{dt} = -[BH3{:}Bax]_{activation}$$

$$\frac{d[Caspase3]}{dt} = -[mitochondrialApoptosis]$$

$$\frac{d[mitophagy]}{dt} = [Mitophagy]_{activation}$$

$$\frac{d[mitochondrialApoptosis]}{dt} = [mitochondrialApoptosis]$$
