# Supplementary figures and images for: Autophagy capacity and sub-mitochondrial heterogeneity shape Bnip3-induced mitophagy regulation of apoptosis
Source: Cell Commun Signal. 2015 Aug 8;13:37. doi: 10.1186/s12964-015-0115-9 (PMC4528699; doi:10.1186/s12964-015-0115-9)

# Supplementary Figure S2

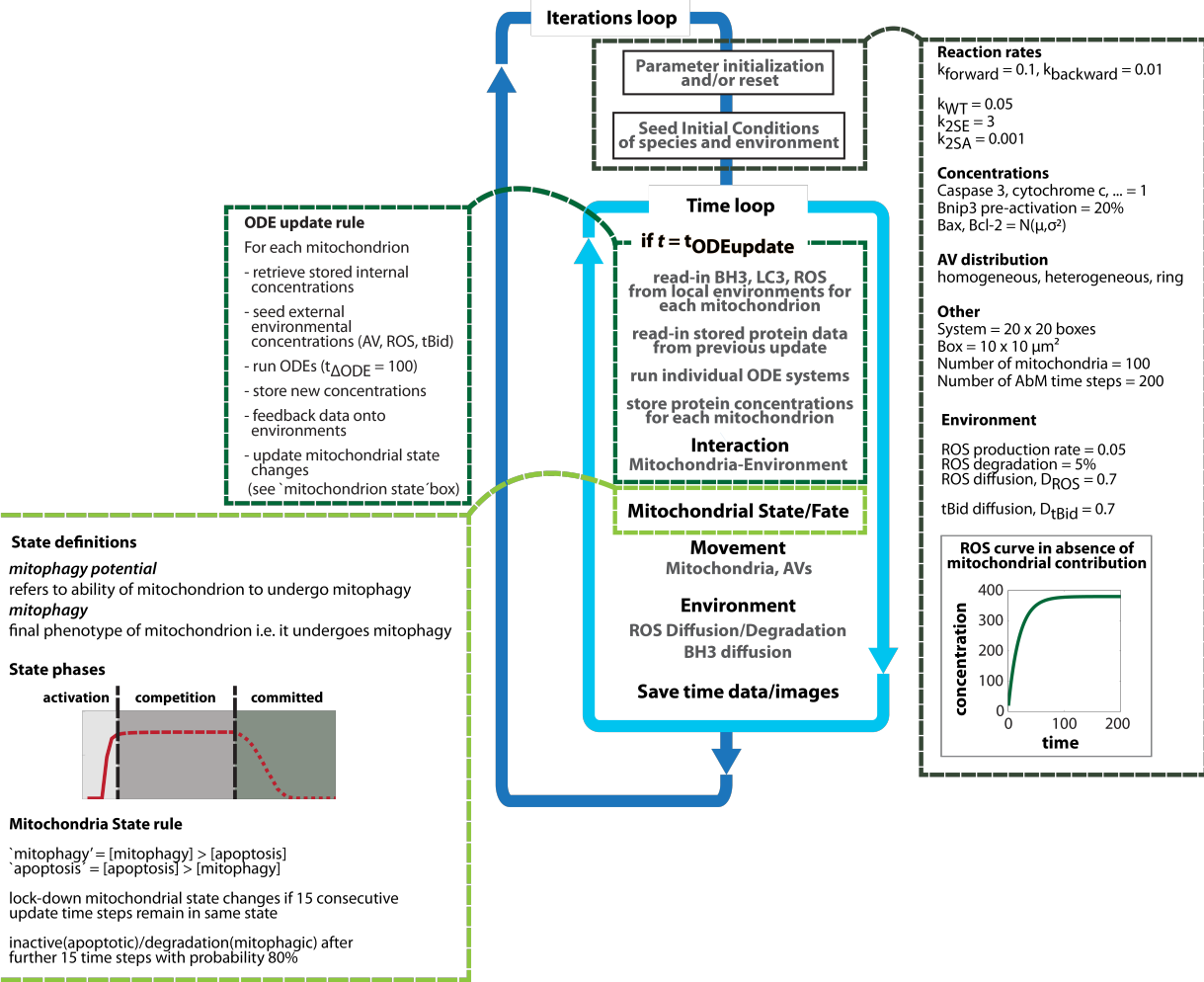

Supplement: Additional file 2: Figure S2. — Flow diagram of time iteration loops for implementation of all update events in the multi-scale model. (Dark blue) Agent-based model updates (light blue) ODE updates for all mitochondria (dashed dark green) ODE update rules (dashed light green) mitochondrial state definitions (dashed gray) parameter values for model. (PDF 928 kb) [file 12964_2015_115_MOESM2_ESM.pdf]

# Supplementary Figure S3

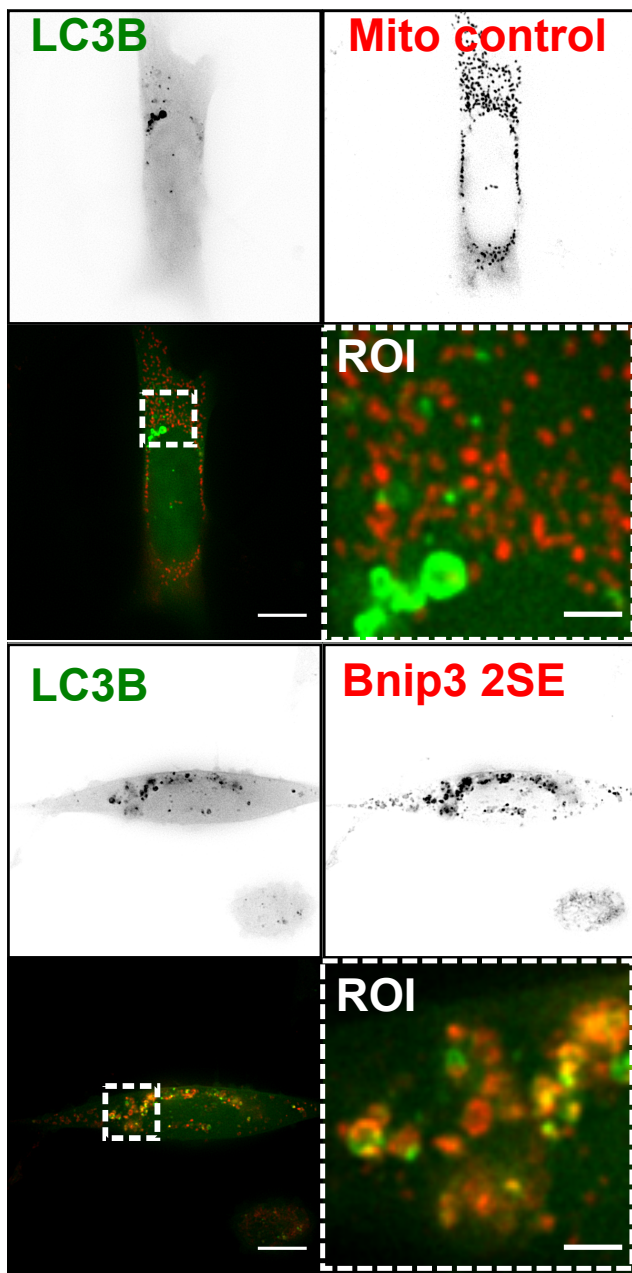

Supplement: Additional file 3: Figure S3. — Experimental validation of two distinct pathways via Bnip3. Bax/Bak DKO cells were co-transfected 24 h with GFP-LC3B and either mito-RFP or RFP-Bnip3 2SE. Bax/Bak DKO cells with apoptosis disabled show no co-localization of autophagosomes (GFP-LC3) with mitochondria (Mito-RFP). Cells expressing active-LIR RFP-Bnip3 2SE mutant show co-localization. (PDF 1788 kb) [file 12964_2015_115_MOESM3_ESM.pdf]

## Supplementary Figure S4

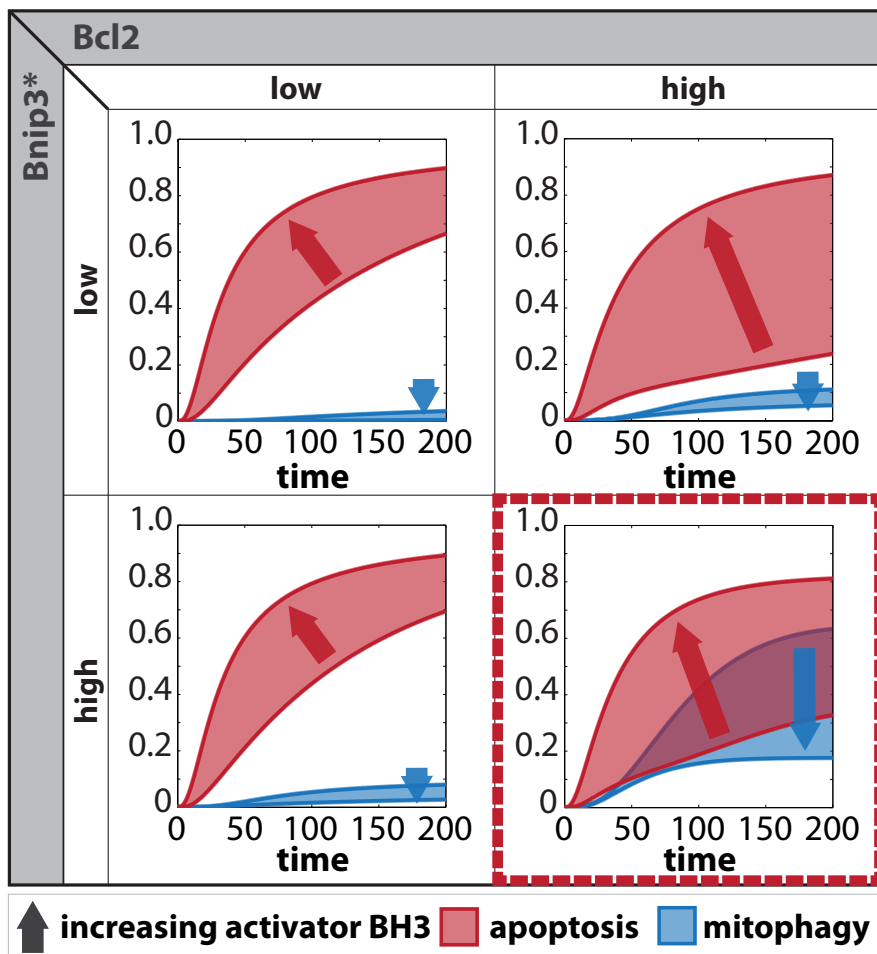

**\* with 20% Bnip3 pre-activation**

Supplement: Additional file 4: Figure S4. — Mitophagy (blue) versus apoptosis (red) activity for different combinations of Bnip3 and Bcl2 levels, as a function of increasing tBid activation (direction of arrow) and 20 % Bnip3 pre-activation. Experimentally observed dual-functionality of Bnip3 only qualitatively reproducible in regime with Bnip3 and Bcl2 simultaneously at high levels; otherwise apoptosis pathway dominates. (PDF 486 kb) [file 12964_2015_115_MOESM4_ESM.pdf]

# Supplementary Figure S5

## A

### Fused phenotype

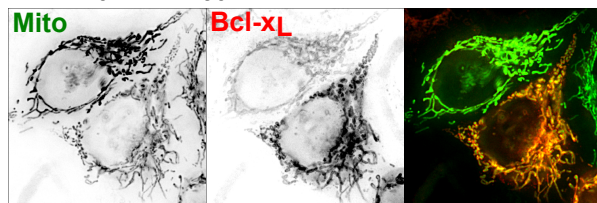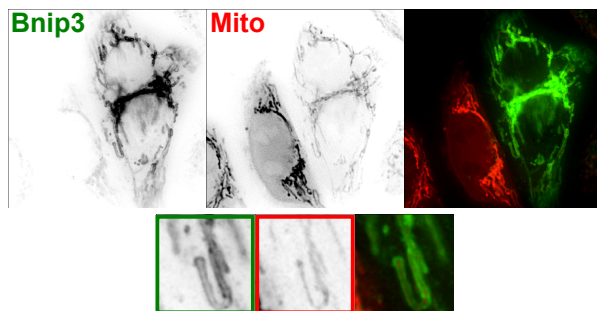

### Fragmented phenotype

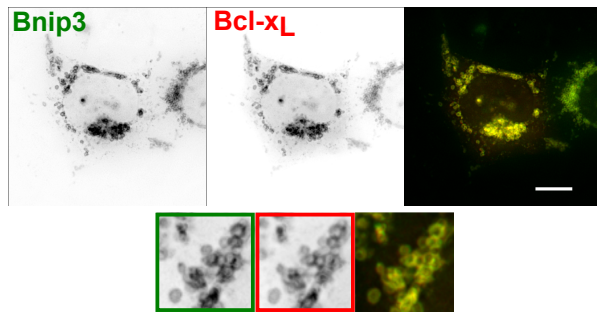

## B

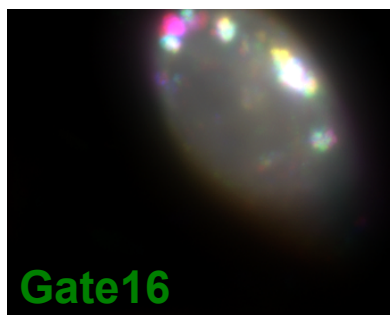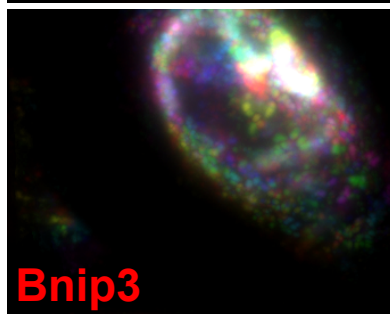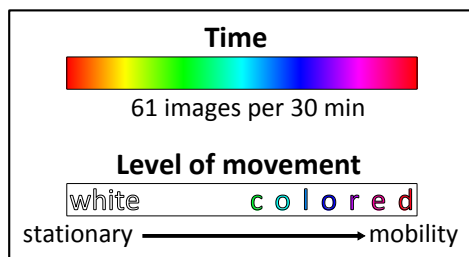

Supplement: Additional file 5: Figure S5. — A Experimental validation of fragmented mitochondria in stressed cells, underlying model assumption of no mitochondrial fission/fusion events. HeLa cells were co-transfected 24 h with indicated combinations of mito-GFP, GFP-Bnip3, and RFP-Bcl-xL. (Fused mitochondria) Individual expression of Bcl-xL (top) or Bnip3 (middle) show elongated mitochondrial structure, (Fragmented mitochondria) Co-expression of Bcl-xL and Bnip3 exhibits highly fragmented mitochondria and co-localization (yellow). B HeLa cells were co-transfected 24 h with GFP-GATE16 and RFP-Bnip3 2SE. Projections of time lapse images for autophagosomes (Gate 16) and mitochondria (Bnip3) depict organelle mobility. After treatment (see Methods), images were taken in 30 s increments for 30 min, and overlaid. The white regions for autophagosomes indicate immobility, while the wide dispersal for mitochondria is indicative of high mobility. (PDF 3157 kb) [file 12964_2015_115_MOESM5_ESM.pdf]

# Supplementary Figure S6

**A**

without tBid

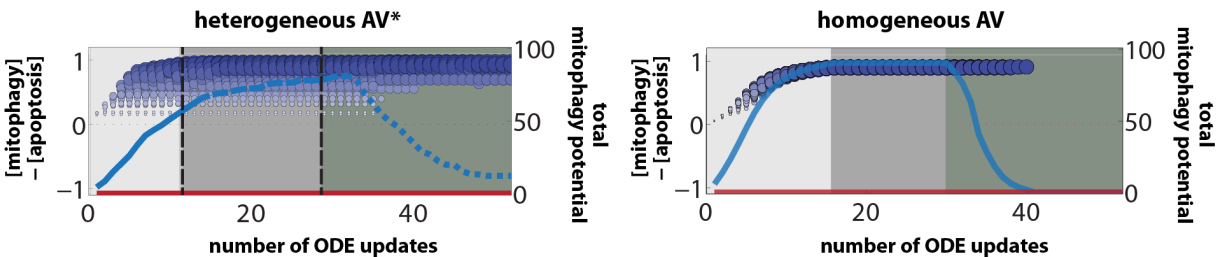

**B**

with tBid

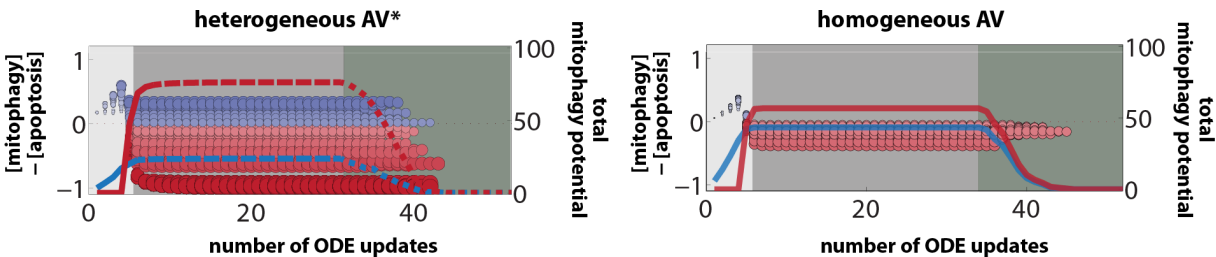

\* Figures from Figure 3A and 3B for comparison

Supplement: Additional file 6: Figure S6. — A Comparison of heterogeneous versus homogeneous AV distribution in a homogeneous mitochondrial population with Bnip3 pre-activation. Scatter points track mitochondrial content individually, with size and color depth indicating level of mitophagy (blue) or apoptosis (red) potential at every time step. Total mitophagic content in mitochondrial population (blue line) indicates the population response. The population exhibits three phases (insets): activation of signaling pathways (light gray), competition to commit to a phenotype (dark gray), and a committed phase during which phenotype is executed (olive). Runs show cell-to-cell variability (blue shaded area) B tBid activation induces some variability due to emergence of heterogeneous ROS environment. Total mitophagic and apoptosis potential curves were averaged of 50 sample runs. (PDF 239 kb) [file 12964_2015_115_MOESM6_ESM.pdf]

# Supplementary Figure S14

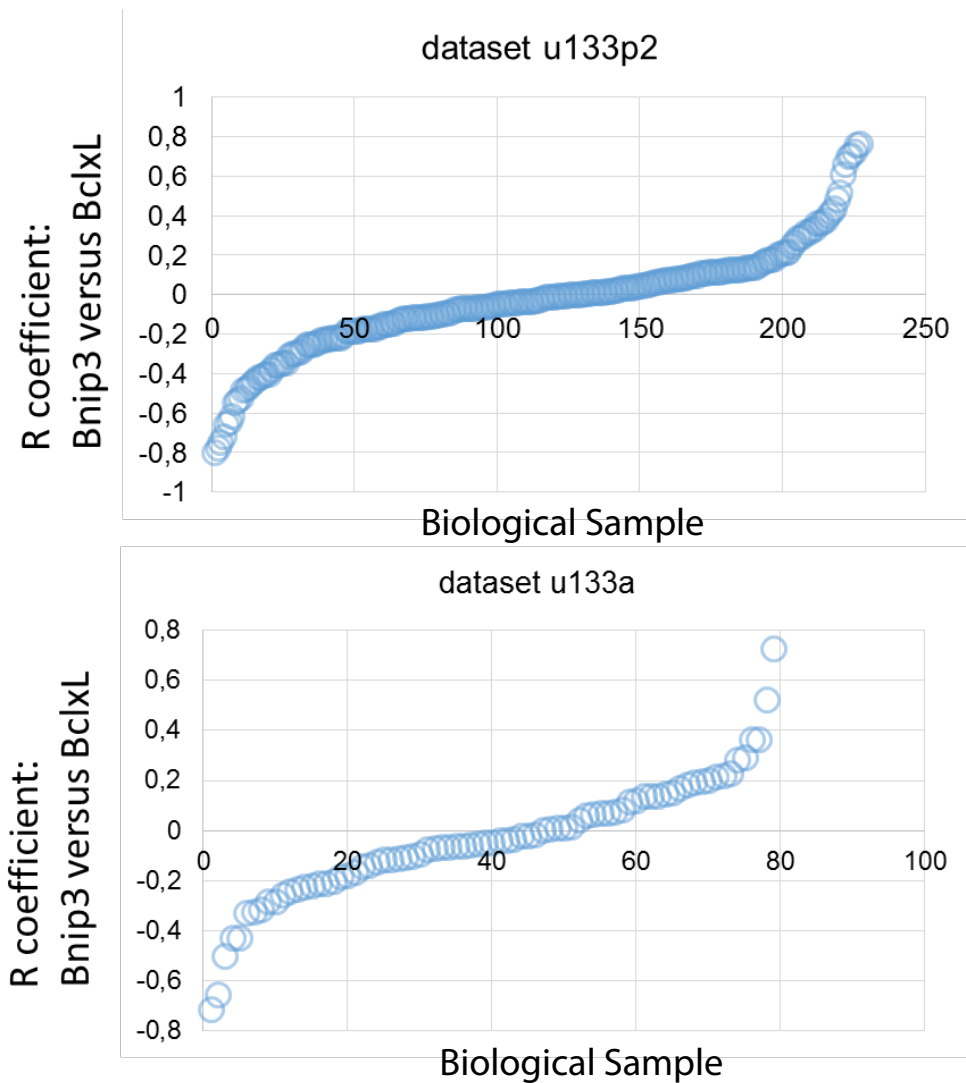

Supplement: Additional file 14: Figure S14. — Dataset of several disease and non-disease states show positive correlation of Bcl-xL and Bnip3 expression. Using the R2: Genomics Analysis and Visualization Platform (http://hgserver1.amc.nl/cgi-bin/r2/main.cgi), a 2D gene overview was performed across all datasets, which include mRNA expression from diseased and normal tissues. The R coefficients for datasets 133a and u133p2 are reported. (PDF 122 kb) [file 12964_2015_115_MOESM14_ESM.pdf]
