# Supplementary material for: Autophagy capacity and sub-mitochondrial heterogeneity shape Bnip3-induced mitophagy regulation of apoptosis
Source: Cell Commun Signal. 2015 Aug 8;13:37. doi: 10.1186/s12964-015-0115-9 (PMC4528699; doi:10.1186/s12964-015-0115-9)
Supplement: Additional file 7: Figure S7. — ROS degradation versus production comparison. Keeping ROS degradation rate constant, variation in non-mitochondrial ROS production rates in Bnip3 WT with A 5-fold decrease compared to control in B and C 4-fold increase. Results show mitochondrial mitophagy decisions (blue histograms) and consequent apoptosis activity indicated by cytochrome c release (red). Simulations of varying ROS degradation rates show little impact on D mitophagy (blue histograms) and E cytochrome c release. Sample size was 50 runs for each condition. Tables show statistics (at t = 100). (PDF 281 kb) [file 12964_2015_115_MOESM7_ESM.pdf]

Supplementary Figure S7

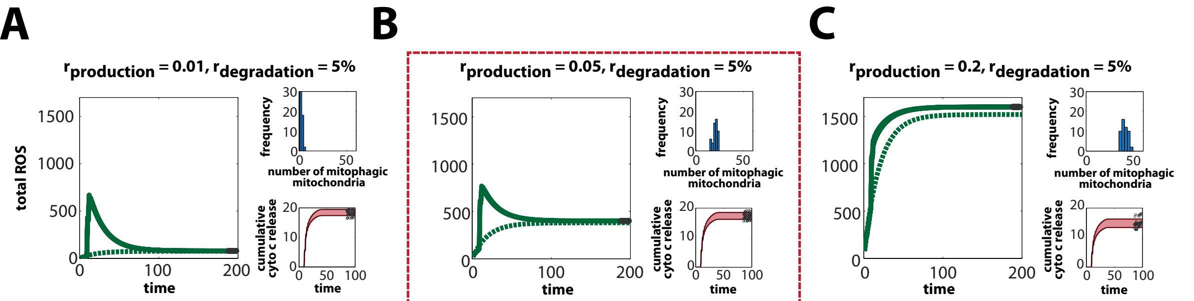

	at t = 200	mean	s.d.	c_v
0.01	mitophagy	2.24	1.46	0.65
	cytochrome c	18.43	1.03	0.06

	at t = 200	mean	s.d.	c_v
0.05	mitophagy	20.36	2.72	0.13
	cytochrome c	17.36	1.15	0.07

	at t = 200	mean	s.d.	c_v
0.2	mitophagy	40.64	3.79	0.09
	cytochrome c	14.86	1.42	0.10

tBid activation at t = 5 for all simulations

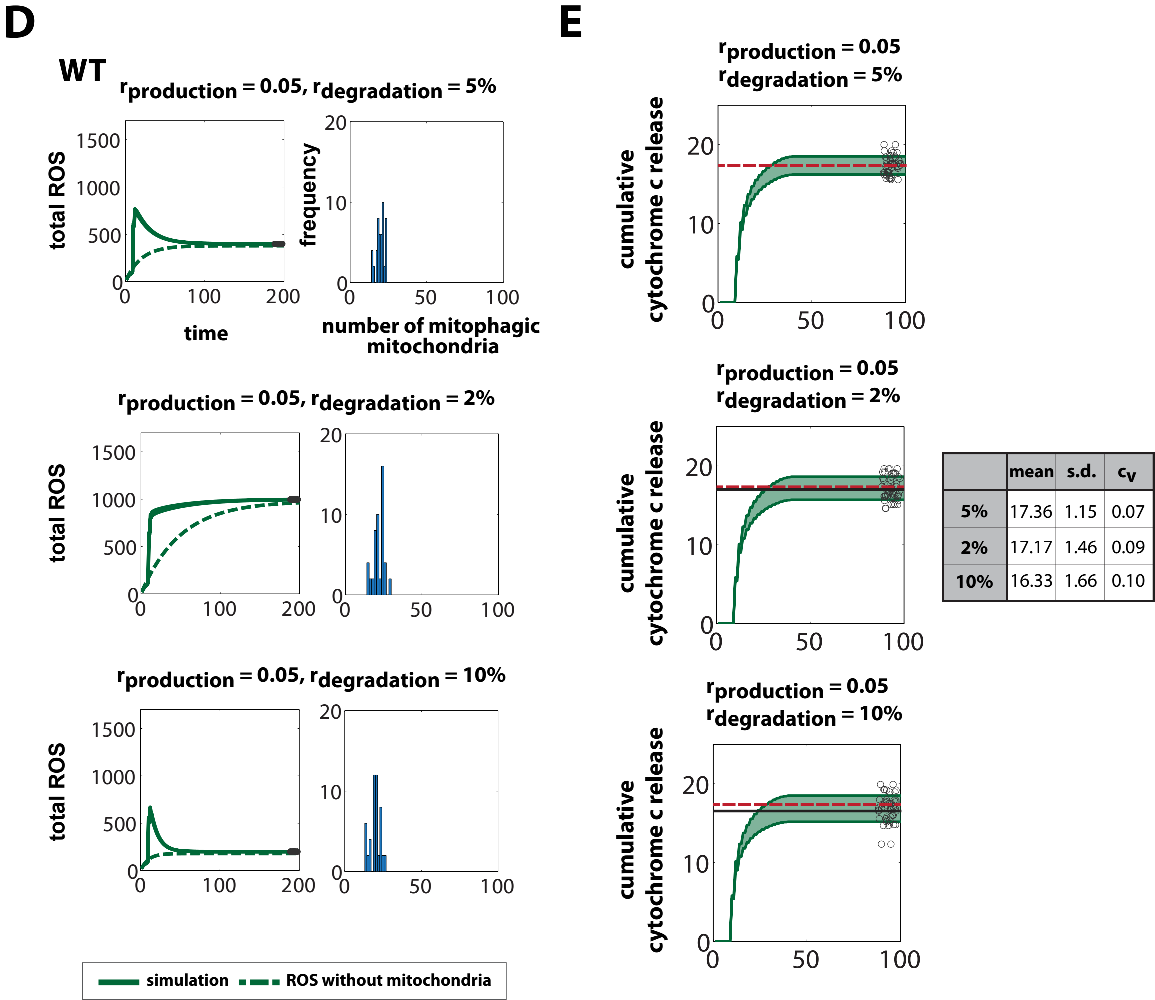

	mean	s.d.	c_v
5%	17.36	1.15	0.07
2%	17.17	1.46	0.09
10%	16.33	1.66	0.10
