# Supplementary material for: Autophagy capacity and sub-mitochondrial heterogeneity shape Bnip3-induced mitophagy regulation of apoptosis
Source: Cell Commun Signal. 2015 Aug 8;13:37. doi: 10.1186/s12964-015-0115-9 (PMC4528699; doi:10.1186/s12964-015-0115-9)
Supplement: Additional file 8: Figure S8. — Statistics for Fig. 4a. (PDF 338 kb) [file 12964_2015_115_MOESM8_ESM.pdf]

Supplementary Figure S8

A

Statistics for Figure 4A (left)

Capan1

	FM	FM + Baf
mean	0.0634	0.1420
s.d.	0.0471	0.0699
median	0.0543	0.1411
CV	0.7419	0.4925
p-value	→	p < 0.001

Panc1

	FM	FM + Baf
mean	0.0547	0.0831
s.d.	0.0504	0.0588
median	0.0452	0.0827
CV	0.9226	0.7076
p-value	→	p < 0.001

HPDE

	FM	FM + Baf
mean	0.0945	0.1237
s.d.	0.7460	0.0733
median	0.0906	0.1179
CV	0.7888	0.5930
p-value	→	p < 0.001

HeLa

	FM	FM + Baf
mean	0.0336	0.1069
s.d.	0.0316	0.0599
median	0.0252	0.1058
CV	0.9405	0.5606
p-value	→	p < 0.001

MCF7

	FM	FM + Baf
mean	0.0375	0.1603
s.d.	0.0367	0.0679
median	0.0275	0.1612
CV	0.9804	0.4234
p-value	→	p < 0.001

MCF10A

	FM	FM + Baf
mean	0.0808	0.1210
s.d.	0.0562	0.0602
median	0.0721	0.1212
CV	0.6948	0.4974
p-value	→	p < 0.001

B

Statistics for Figure 4A (right)

MCF7

	FM	FM + Baf	Rad0001	Rad0001 + Baf
mean	0.0085	0.0397	0.0209	0.0704
s.d.	0.0157	0.0415	0.0286	0.0567
median	0.0032	0.0275	0.0109	0.0570
CV	1.8405	1.0453	1.3673	0.8061
p-value	→	p < 0.001	→	p < 0.001

HPDE

	FM	FM + Baf	Rad0001	Rad0001 + Baf
mean	0.0099	0.0698	0.0156	0.1083
s.d.	0.0179	0.0458	0.0213	0.0597
median	0.0041	0.0653	0.0088	0.1084
CV	1.8167	0.6559	1.3631	0.5510
p-value	→	p < 0.001	→	p < 0.001
