# Supplementary material for: Autophagy capacity and sub-mitochondrial heterogeneity shape Bnip3-induced mitophagy regulation of apoptosis
Source: Cell Commun Signal. 2015 Aug 8;13:37. doi: 10.1186/s12964-015-0115-9 (PMC4528699; doi:10.1186/s12964-015-0115-9)

Supplementary Figure S9

A

Statistics for Figure 4C

Bnip3 mutant \ AV		AV = 20			AV = 75			AV = 100			AV = 200			homogeneous		
		mean	s.d.	c_v	mean	s.d.	c_v	mean	s.d.	c_v	mean	s.d.	c_v	mean	s.d.	c_v
WT	mitophagy	7.90	2.84	0.36	30.04	5.12	0.17	39.90	3.33	0.08	65.40	3.39	0.05	75.12	3.37	0.04
	total cyto c release	22.63	1.45	0.06	13.81	1.41	0.10	11.31	1.71	0.15	4.75	0.30	0.06	3.03	0.13	0.04
2SE	mitophagy	16.32	3.91	0.24	50.20	5.53	0.11	61.08	4.42	0.07	85.70	3.00	0.04	96.24	1.92	0.02
	total cyto c release	22.56	2.07	0.09	12.22	1.51	0.12	9.50	1.53	0.16	2.89	0.98	0.23	0.81	0.12	0.15
2SA	mitophagy	0	0	—	0.04	0.20	4.95	0.16	0.47	2.92	0.12	0.33	2.74	0	0	—
	total cyto c release	26.64	2.00	0.08	24.12	1.26	0.05	23.44	1.15	0.05	20.41	1.31	0.06	23.83	1.02	0.04

B

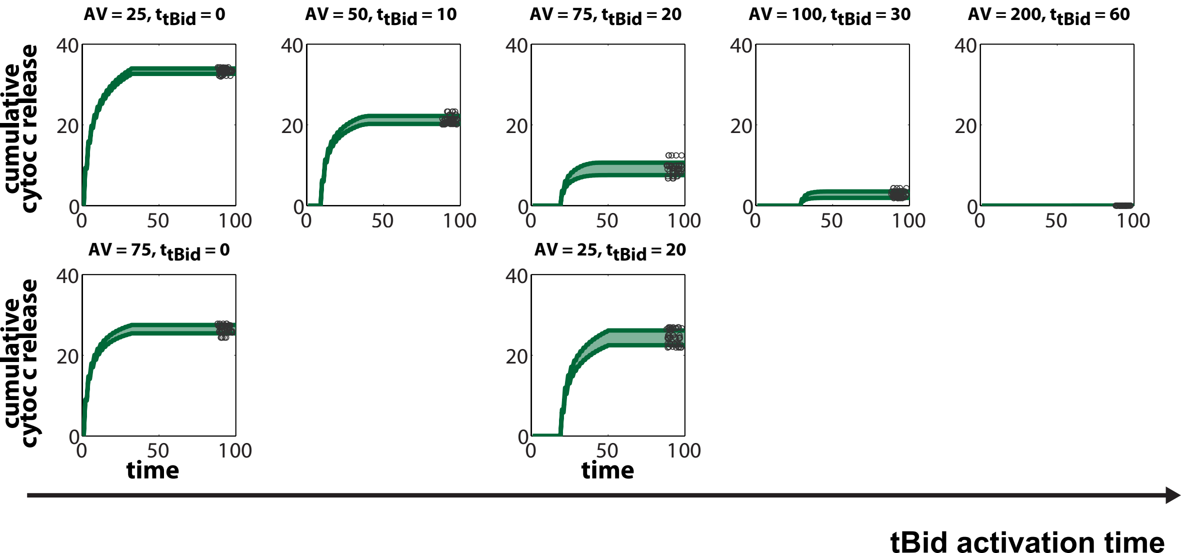

Supplement: Additional file 9: Figure S9. — A Statistics for Fig. 4c B Impact of different combinations of AV level versus tBid activation time on cytochrome c release. Sample size was 50 runs for each condition. (PDF 194 kb) [file 12964_2015_115_MOESM9_ESM.pdf]
