# Supplementary material for: Autophagy capacity and sub-mitochondrial heterogeneity shape Bnip3-induced mitophagy regulation of apoptosis
Source: Cell Commun Signal. 2015 Aug 8;13:37. doi: 10.1186/s12964-015-0115-9 (PMC4528699; doi:10.1186/s12964-015-0115-9)
Supplement: Additional file 10: Figure S10. — Statistics for Fig. 5. (PDF 53 kb) [file 12964_2015_115_MOESM10_ESM.pdf]

Supplementary Figure S10

A

HL-1

	WT	2SE	2SA
mean	0.0381	0.0652	0.0346
s.d.	0.0444	0.0609	0.0411
median	0.0226	0.0504	0.0207
CV	1.1659	0.9345	1.1905
p-value	WT → 2SE: $p < 0.001$; WT → 2SA: $p \approx 0.30$; 2SE → 2SA: $p < 0.001$		

HeLa

	WT	2SE	2SA
mean	0.0710	0.0815	0.0650
s.d.	0.0729	0.0783	0.0699
median	0.0482	0.0585	0.0424
CV	1.0277	0.9603	1.0757
p-value	WT → 2SE: $p < 0.001$; WT → 2SA: $p < 0.001$; 2SE → 2SA: $p < 0.001$		

B

HeLa

	Bnip3 WT	Bnip3 WT + CCCP	Bnip3 2SE	Bnip3 2SE + CCCP
mean	0.0139	0.0312	0.0959	0.2781
s.d.	0.0104	0.0235	0.0802	0.1595
median	0.0110	0.0291	0.0718	0.2160
CV	0.7490	0.7552	0.8358	0.5737
p-value	Bnip3 WT → Bnip3 WT + CCCP: $p = 0.1328$; Bnip3 2SE → Bnip3 2SE + CCCP: $p = 0.0040$; Bnip3 WT → Bnip3 2SE: $p < 0.001$; Bnip3 WT + CCCP → Bnip3 2SE + CCCP: $p < 0.001$			
