# Supplementary material for: Autophagy capacity and sub-mitochondrial heterogeneity shape Bnip3-induced mitophagy regulation of apoptosis
Source: Cell Commun Signal. 2015 Aug 8;13:37. doi: 10.1186/s12964-015-0115-9 (PMC4528699; doi:10.1186/s12964-015-0115-9)
Supplement: Additional file 11: Figure S11. — A Simulations of reducing AV levels three-fold compared to standard levels (AV = 75, Fig. 6b) and no tBid activation. B Comparison of stationary mitochondria (solid) versus dynamic mitochondria (hashed) for increasing radii of AV distribution rings with tBid activation (at AV = 75) compared to heterogeneous AV distribution (red). Sample size was 50 runs for each condition. C Statistics for Fig. 6e. (PDF 477 kb) [file 12964_2015_115_MOESM11_ESM.pdf]

Supplementary Figure S11

A

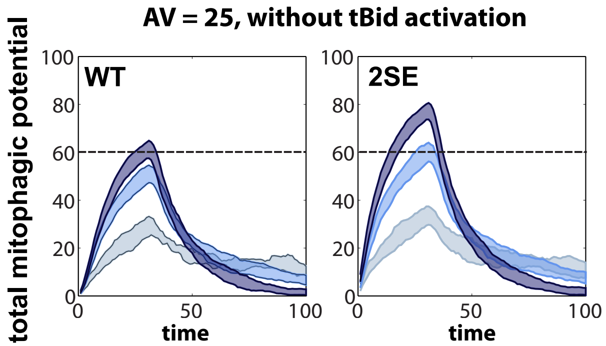

B

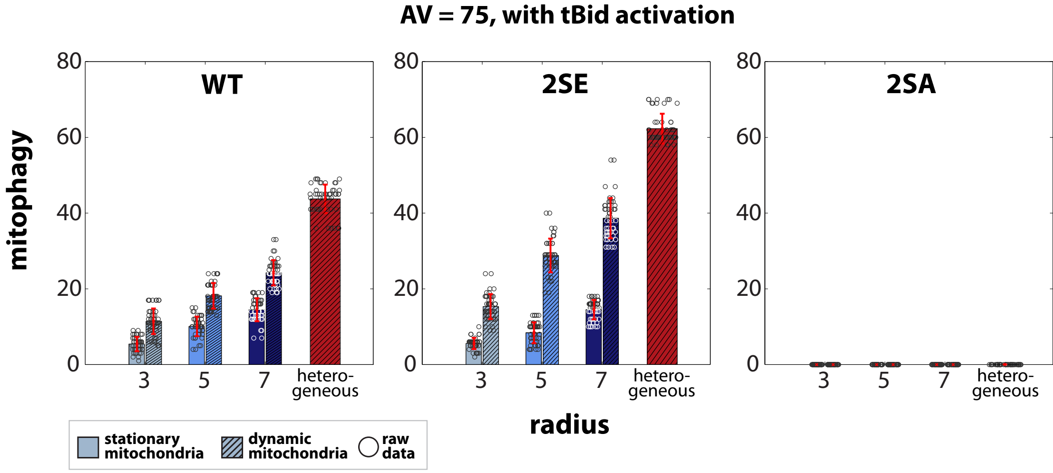

C

	pcDNA3.1	pcDNA3-RILP WT	pcDNA3-ΔRILP	DN DRP1
mean	0.0843	0.1174	0.1698	0.0246
s.d.	0.0522	0.0514	0.0897	0.0145
median	0.0776	0.1166	0.1798	0.0282
CV	0.6187	0.4375	0.5280	0.5887
p-value	pcDNA3.1 → pcDNA3-RILP WT: $p = 0.1364$; pcDNA3.1 → pcDNA3-ΔRILP: $p = 0.0125$; pcDNA3.1 → DN DRP1: $p < 0.0001$; pcDNA3-RILP WT → pcDNA3-ΔRILP: $p = 0.1181$			
