# Supplementary material for: Autophagy capacity and sub-mitochondrial heterogeneity shape Bnip3-induced mitophagy regulation of apoptosis
Source: Cell Commun Signal. 2015 Aug 8;13:37. doi: 10.1186/s12964-015-0115-9 (PMC4528699; doi:10.1186/s12964-015-0115-9)
Supplement: Additional file 12: Figure S12. — Comparison of mitochondrial versus AV mobility: movement of either subcellular organelles (middle two groups) compared to immobile system (left group), and mobility of both species (right group), with corresponding total cytochrome c release behavior. Table shows statistics for each condition with sample size of 50 runs each. (PDF 483 kb) [file 12964_2015_115_MOESM12_ESM.pdf]

Supplementary Figure S12

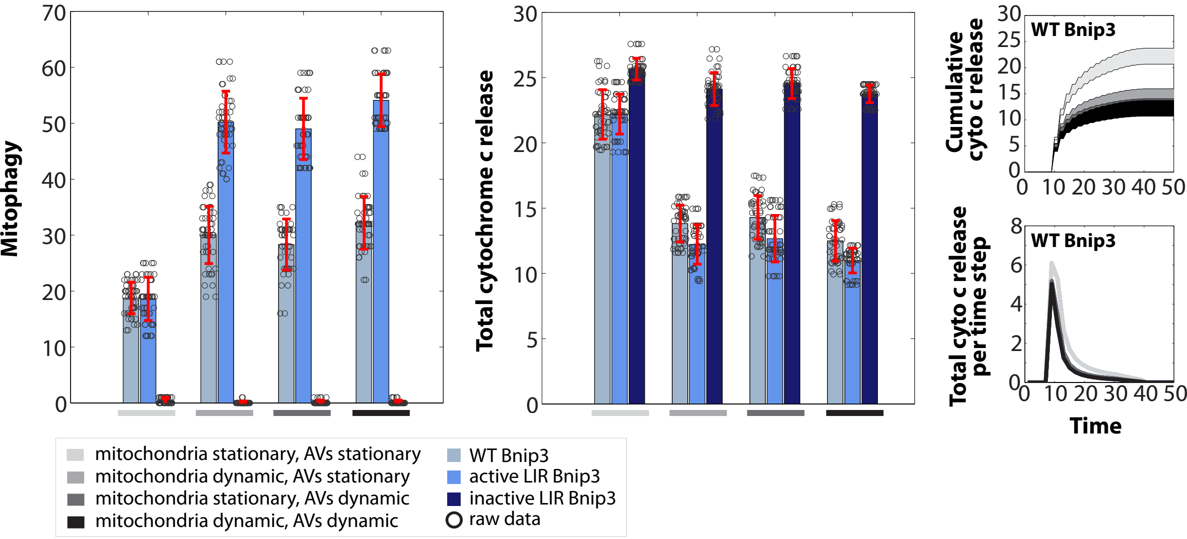

Bnip3 mutant \ dynamics		mitochondria stationary AV stationary			mitochondria dynamic AV stationary			mitochondria stationary AV dynamic			mitochondria dynamic AV dynamic		
		mean	s.d.	c_v	mean	s.d.	c_v	mean	s.d.	c_v	mean	s.d.	c_v
WT	mitophagy	18.76	2.82	0.15	30.04	5.12	0.17	28.32	4.56	0.16	32.20	4.69	0.15
	total cyto c release	22.18	1.90	0.09	13.81	1.41	0.10	14.29	1.67	0.12	12.49	1.55	0.12
2SE	mitophagy	18.60	3.87	0.21	50.20	5.53	0.11	49.00	5.48	0.11	54.10	4.70	0.09
	total cyto c release	22.20	1.53	0.07	12.22	1.51	0.12	12.66	1.78	0.14	10.98	0.95	0.09
2SA	mitophagy	0.40	0.49	1.24	0.04	0.20	4.95	0.01	0.30	3.03	0.10	0.03	3.03
	total cyto c release	25.65	0.83	0.03	24.12	1.26	0.05	24.54	1.15	0.05	23.75	0.68	0.03
