# Supplementary material for: Autophagy capacity and sub-mitochondrial heterogeneity shape Bnip3-induced mitophagy regulation of apoptosis
Source: Cell Commun Signal. 2015 Aug 8;13:37. doi: 10.1186/s12964-015-0115-9 (PMC4528699; doi:10.1186/s12964-015-0115-9)
Supplement: Additional file 13: Figure S13. — Statistics for Figs. 7a and 7b. (PDF 45 kb) [file 12964_2015_115_MOESM13_ESM.pdf]

Supplementary Figure S13

A

Statistics for Figure 7A

Bnip3 mutant \ heterogeneity		control			s.d. = 0.1			s.d. = 0.3			s.d. = 0.6		
		mean	s.d.	c_v	mean	s.d.	c_v	mean	s.d.	c_v	mean	s.d.	c_v
WT	mitophagy	20.50	2.64	0.13	19.80	5.32	0.27	23.20	3.61	0.16	30.00	5.68	0.19
	total cyto c release	16.96	0.96	0.06	17.06	1.67	0.10	15.18	1.83	0.12	14.37	1.43	0.10
2SE	mitophagy	51.80	8.58	0.17	51.64	4.16	0.08	56.20	4.14	0.07	56.68	4.58	0.08
	total cyto c release	14.14	2.31	0.16	13.57	1.28	0.09	11.97	1.25	0.10	12.12	1.57	0.13
2SA	mitophagy	0	0	-	0	0	-	0	0	-	1.40	1.18	0.84
	total cyto c release	32.29	0.46	0.01	28.49	0.99	0.03	24.64	1.08	0.04	23.16	1.32	0.06

B

Statistics for Figure 7B

Bnip3 mutant \ mean		$mean_{Bcl2}$ = 0.5			$mean_{Bcl2}$ = 1.0			$mean_{Bcl2}$ = 2.0		
		mean	s.d.	c_v	mean	s.d.	c_v	mean	s.d.	c_v
WT	mitophagy	1.72	1.05	0.61	21.16	4.29	0.20	31.92	4.06	0.13
	total cyto c release	19.14	1.72	0.09	17.25	1.24	0.07	14.52	1.51	0.10
2SE	mitophagy	17.90	3.82	0.21	54.30	4.36	0.08	52.90	5.35	0.10
	total cyto c release	16.76	0.79	0.05	13.74	1.19	0.09	12.61	1.48	0.12
2SA	mitophagy	0	0	-	0	0	-	0	0	-
	total cyto c release	34.29	0.33	0.01	32.23	0.63	0.02	29.23	0.58	0.02
